# Non-Infectious Causes for Elevated Procalcitonin

**DOI:** 10.3390/medicina62030464

**Published:** 2026-02-28

**Authors:** Stefan Lucian Popa, Victor Incze, Abdulrahman Ismaiel, Teodora Surdea-Blaga, Simona Grad, Daria Claudia Turtoi, Darius-Stefan Amarie, Liliana David, Vlad Dumitru Brata, Daniel Corneliu Leucuta, Ahmed Abdelghafar, Claudia Diana Gherman, Mihai Razvan Zahan, Dinu Iuliu Dumitrascu

**Affiliations:** 1 2nd Medical Department, “Iuliu Hatieganu” University of Medicine and Pharmacy, 400000 Cluj-Napoca, Romania; popa.stefan@umfcluj.ro (S.L.P.); abdulrahman.ismaiel@yahoo.com (A.I.); dora_blaga@yahoo.com (T.S.-B.); costinsimona_m@yahoo.com (S.G.); lilidavid2007@yahoo.com (L.D.); zahan_razvan@yahoo.com (M.R.Z.); 2 Faculty of Medicine, “Iuliu Hatieganu” University of Medicine and Pharmacy, 400000 Cluj-Napoca, Romania; dariusamarie27@gmail.com (D.-S.A.); ahmedabdelghafar2002@gmail.com (A.A.); 3 Department of Radiology, County University Emergency Hospital, 400006 Cluj-Napoca, Romania; turtoidariaclaudia@gmail.com; 4 Department of Gastroenterology, Regional Institute of Gastroenterology and Hepatology “Prof. Dr. Octavian Fodor”, 400394 Cluj-Napoca, Romania; brata_vlad@yahoo.com; 5 Department of Medical Informatics and Biostatistics, “Iuliu Hatieganu” University of Medicine and Pharmacy, 400349 Cluj-Napoca, Romania; dleucuta@umfcluj.ro; 6 Department of Surgery-Practical Abilities, “Iuliu Hațieganu” University of Medicine and Pharmacy, 400337 Cluj-Napoca, Romania; gherman.claudia@umfcluj.ro; 7 Department of Anatomy, “Iuliu Hatieganu” University of Medicine and Pharmacy, 400000 Cluj-Napoca, Romania; d.dumitrascu@yahoo.com

**Keywords:** procalcitonin, non-infectious inflammation, systemic inflammatory response, trauma, surgery, shock, acute pancreatitis, malignancy, renal failure, transplantation

## Abstract

*Background and Objectives*: Procalcitonin (PCT) is widely used to support the diagnosis of bacterial infection and sepsis, yet clinically relevant elevations also occur in multiple non-infectious conditions. This systematic review aimed to synthesize human evidence on non-infectious causes of elevated PCT and to summarize proposed pathophysiological mechanisms, with the goal of supporting context-based interpretation in clinical practice. *Materials and Methods*: A systematic search of PubMed/MEDLINE, Embase, Web of Science, and Scopus was performed from inception to 31 July 2025. Human studies published in English reporting quantitative PCT values in non-infectious contexts were eligible (observational studies, clinical trials, and case series with ≥5 patients). *Results*: Seventy-six unique studies were included. Evidence was organized across systemic inflammatory responses, cardiovascular pathology, nephrological disorders and renal replacement therapy, pulmonary diseases, gastrointestinal and hepatopancreatic diseases, autoimmune and rheumatologic conditions, neurologic and ophthalmologic conditions, onco-hematologic disorders, surgery, traumatology and transplanted patients. Across conditions, non-infectious PCT elevations were variable and frequently overlapped with ranges reported in bacterial infection, particularly in settings characterized by severe sterile inflammation and tissue injury (e.g., major surgery, trauma, shock, pancreatitis, and burns), as well as in selected malignancies with tumor-associated PCT production. *Conclusions*: Elevated PCT is not synonymous with bacterial infection. Interpretation should emphasize clinical context, timing, and serial trends rather than isolated thresholds, especially in high-acuity settings with strong non-infectious inflammatory stimuli. Standardized reporting of assays and sampling time points and condition-specific kinetic data are needed to refine diagnostic and stewardship algorithms.

## 1. Introduction

Procalcitonin (PCT) is a 116–amino acid peptide initially identified by Le Moullec et al. in 1984 [1]. PCT was first identified as a biomarker of bacterial infection in 1993, when elevated levels of calcitonin-like immunoreactivity were detected in the circulation of patients with extra-thyroidal diseases [2] and later in 1994 when injecting healthy volunteers with endotoxin raised their PCT values [3].

Under physiological conditions, pre-procalcitonin is synthesized primarily by thyroid C cells. This precursor peptide is subsequently processed into procalcitonin through endopeptidase-mediated cleavage of a 25–amino acid signal peptide. Further enzymatic conversion by prohormone convertases yields calcitonin, a 32–amino acid hormone that plays a key role in the regulation of serum calcium homeostasis [4].

Under normal physiological conditions, circulating procalcitonin concentrations remain very low, typically below 0.05 ng/mL. In contrast, exposure to systemic inflammatory stimuli such as circulating endotoxins or proinflammatory cytokines including interleukin-6 (IL-6), tumor necrosis factor-α (TNF-α), and interleukin-1β (IL-1β) can induce a marked upregulation of PCT synthesis, with increases of up to 100- to 1000-fold across multiple tissues [5]. Extra-thyroidal production of procalcitonin occurs in multiple organs, including the liver, pancreas, kidneys, lungs, intestines, and circulating leukocytes. Importantly, PCT synthesis in these tissues remains largely suppressed under non-infectious conditions [6]. In contrast, cytokines released during viral infections, notably interferon-gamma (IFN-γ), have been shown to downregulate PCT synthesis [7]. However, PCT should not be relied upon as the sole indicator for initiating antimicrobial therapy and should be integrated within the broader clinical context [8,9].

Serum PCT levels have been observed to rise within 6 to 12 h after the onset of a bacterial infection and to increase progressively during the first 2 to 4 h following the development of sepsis [5,10]. PCT has a half-life of approximately 20 to 24 h. Consequently, with effective host immune responses and appropriate antibiotic therapy, serum PCT concentrations typically decline by about 50% within 24 h [6]. The majority of studies indicate that PCT demonstrates clinical relevance at serum levels between 0.1 and 0.5 ng/mL, while PCT concentrations below 0.1 ng/mL have been reported to possess a high negative predictive value for ruling out bacterial infections. [6,11].

Procalcitonin has become firmly established as a biomarker of substantial clinical importance in the assessment of systemic inflammatory responses, particularly in the differentiation of bacterial infections from inflammation of non-infectious origin. Initially characterized for its relative specificity in the context of bacterial sepsis, circulating procalcitonin concentrations have increasingly been incorporated into evidence-based diagnostic algorithms and antimicrobial stewardship frameworks [12]. Within these clinical pathways, elevated procalcitonin values frequently serve as a trigger for the early initiation of antibacterial therapy, assist in stratifying the severity of systemic infection, and guide decisions regarding treatment duration [12]. As a consequence, procalcitonin now occupies a central role in contemporary approaches to managing suspected infection, where its kinetics and threshold values are often used to support high-stakes decisions in emergency, critical care, and inpatient settings [12].

Although the diagnostic utility of procalcitonin for identifying bacterial etiology has been repeatedly validated, a growing body of evidence demonstrates that procalcitonin elevation cannot be regarded as pathognomonic for infection [13]. Rather, substantial increases in circulating procalcitonin have been observed across a wide spectrum of non-infectious conditions. These include severe blunt and penetrating trauma, major surgical interventions associated with extensive tissue injury, cardiogenic shock with attendant hypoperfusion, acute pancreatitis characterized by intense systemic inflammatory activation, and various malignant processes capable of inducing paraneoplastic cytokine release [12,13]. In such settings, the pathways leading to procalcitonin induction are heterogeneous and involve complex interactions among the innate immune system, endocrine and neuroendocrine stress responses, and cytokine-mediated signalling networks [12,13]. Interleukin-6, tumour necrosis factor-alpha, and other pro-inflammatory mediators are believed to modulate extrathyroidal procalcitonin expression during severe non-infectious insults, thereby mimicking the biomarker profile typically associated with bacterial infections.

Recognition of these non-infectious mechanisms is of considerable clinical importance. Failure to distinguish infectious from sterile causes of elevated procalcitonin may result in diagnostic misclassification and inappropriate initiation or escalation of antibiotic therapy. Such misinterpretation carries meaningful consequences, including avoidable adverse drug reactions, prolongation of hospital stay, disruption of microbiome homeostasis, and contribution to the already accelerating problem of antimicrobial resistance. Consequently, the interpretation of procalcitonin values must always be contextualized within the full clinical scenario, considering the timing of measurement, the presence of comorbidities such as trauma, shock, or malignancy, and the dynamics of the patient’s overall inflammatory profile [14].

Despite a substantial increase in published reports describing non-infectious elevations in procalcitonin, the current literature remains fragmented and lacks a unified synthesis across clinical conditions. Individual studies often focus on single disease entities or specific patient populations, which limits the generalizability of their findings. Moreover, the pathophysiological mechanisms proposed to account for extraneous procalcitonin elevation have not been consistently evaluated across studies, leading to variability in reported mechanisms, biomarker ranges, and diagnostic implications [14]. These gaps underscore the need for a systematic and comprehensive evaluation of the non-infectious determinants of procalcitonin elevation.

The present systematic review was therefore designed to consolidate and critically appraise existing evidence regarding non-infectious conditions associated with elevated procalcitonin levels. Its objectives are to categorize the major clinical contexts in which procalcitonin rises in the absence of infection, to elucidate the underlying biological mechanisms contributing to these elevations, and to delineate the diagnostic scenarios in which procalcitonin may be misleading if interpreted without adequate clinical nuance. Through a rigorous synthesis of available data, this review aims to refine the interpretive framework for procalcitonin and to support a more informed, precise, and judicious use of this biomarker in both clinical practice and future research.

## 2. Materials and Methods

This systematic review was conducted and reported in accordance with the Preferred Reporting Items for Systematic Reviews and Meta-Analyses (PRISMA) guidelines and was registered in the International Platform of Registered Systematic Review and Me-ta-analysis Protocols (INPLASY) (Registration ID: INPLASY202620023) [15]. The objective was to identify, critically appraise, and synthesize evidence regarding non-infectious clinical conditions associated with elevated procalcitonin (PCT) levels in humans, and to summarize the pathophysiological mechanisms proposed to explain these elevations.

### 2.1. Eligibility Criteria

Studies were eligible if they met all of the following criteria: (i) conducted in human participants; (ii) published in English; (iii) reported quantitative PCT measurements (e.g., absolute values, ranges, or summary statistics); and (iv) investigated non-infectious contexts in which PCT may be elevated, including (but not limited to) trauma, surgery, shock, pancreatitis, burns, malignancy, and autoimmune/inflammatory diseases. Eligible study designs included observational studies (prospective or retrospective cohort studies and case–control studies), clinical trials, and case series with ≥5 patients.

Studies were excluded if they: (i) focused exclusively on infectious etiologies of PCT elevation; (ii) were conducted in animals or in vitro without direct clinical correlation in humans; (iii) were reviews, editorials, letters, or conference abstracts without original patient-level data; or (iv) did not provide sufficient information to interpret PCT results (e.g., absence of measurement details and/or sampling time points).

### 2.2. Search Strategy and Study Selection

A comprehensive literature search was performed in the following electronic databases: PubMed/MEDLINE, Embase, Web of Science, and Scopus. All databases were searched from inception through 31 July 2025 (final search date: 31 July 2025). Searches were limited to human studies published in English. In addition, the reference lists of eligible studies and relevant review articles were screened to identify additional potentially eligible records.

The search strategy combined controlled vocabulary terms (e.g., MeSH/Emtree) and free-text keywords related to PCT and non-infectious conditions. Search terms included combinations of: procalcitonin, PCT, non-infectious, trauma, surgery, shock, pancreatitis, burns, malignancy, autoimmune disease, and inflammation. Database-specific syntax and subject headings were adapted for each platform. The full electronic search strategies for all databases (including Boolean operators, field tags, and limits) are provided in the Supplementary Material (Supplementary File S1).

Records retrieved from the searches were collated and duplicates were removed prior to screening. Two reviewers independently screened titles and abstracts for potential eligibility. Full-text articles were obtained for all records deemed potentially eligible and for those in which eligibility could not be determined from the title/abstract alone. Full texts were assessed independently by the same two reviewers against the predefined inclusion and exclusion criteria. Reasons for exclusion were recorded at each stage.

At full-text review, the most common reasons for exclusion were lack of quantitative PCT data, infectious-only study populations, and insufficient information on sampling/measurement to interpret PCT kinetics in a non-infectious context.

**Figure 1:**
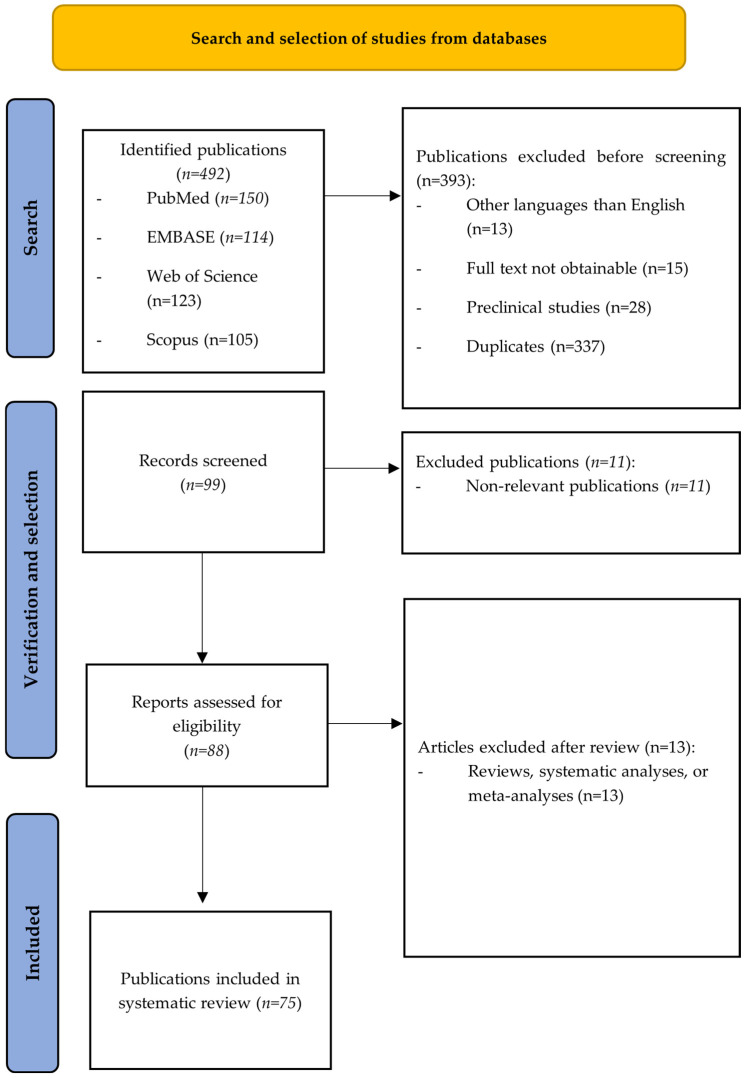
PRISMA flow diagram of study identification, screening, eligibility assessment, and inclusion.

Discrepancies at any stage were resolved through discussion and, when necessary, consultation with a third reviewer. The study selection process is detailed in Figure 1.

### 2.3. Data Extraction and Data Items

Data extraction was performed using a standardized, pilot-tested form. For each included study, the following information was collected: (i) publication details (first author, year, country); (ii) study design and setting; (iii) sample size; (iv) participant characteristics (age, sex, clinical condition); (v) details of PCT assessment (assay or analytical method when reported, sampling time points relative to the clinical event, and reported PCT values/summary statistics); (vi) the non-infectious etiology associated with elevated PCT; (vii) mechanistic explanations proposed by the study authors; and (viii) clinical outcomes when available (e.g., severity measures, complications, mortality, length of stay). Two reviewers extracted data independently and cross-checked all entries. Any disagreements were resolved by consensus, with adjudication by a third reviewer when required. Where studies reported multiple time points, all clinically relevant time points were recorded to capture PCT kinetics.

Methodological quality (risk of bias) was assessed at the study level using design-appropriate tools. The Newcastle–Ottawa Scale (NOS) was applied to cohort and case–control studies, and the Joanna Briggs Institute (JBI) critical appraisal checklist was applied to case series. Each study was rated independently by two reviewers. Disagreements were resolved through discussion and, if needed, third-party adjudication. Studies were categorized as high, moderate, or low methodological quality according to the scoring guidance of the respective instruments.

### 2.4. Data Synthesis

Given substantial clinical and methodological heterogeneity across included studies—particularly with respect to study design, patient populations, underlying non-infectious etiologies, PCT assays, and sampling time points—a narrative synthesis was performed. Findings were organized into clinically meaningful categories (trauma, surgery, shock, pancreatitis, burns, malignancy, autoimmune/inflammatory diseases). Where reported, PCT values were summarized descriptively (e.g., ranges, medians, means) and interpreted in relation to timing and clinical context. Proposed mechanistic pathways underlying non-infectious PCT elevation were extracted and synthesized across studies. A quantitative meta-analysis was not undertaken due to heterogeneity and inconsistent reporting of effect measures. Supplementary Tables were used to summarize study characteristics and PCT findings by clinical domain, alongside timing of measurement and mechanistic interpretations, to enhance comparability across conditions.

## 3. Results

A total of 75 unique studies were included in the qualitative synthesis. The evidence was organized into clinically coherent domains reflecting the major non-infectious contexts in which procalcitonin (PCT) elevations have been reported. Specifically, the Section 3 summarizes findings across systemic inflammatory responses (*n* = 7 studies), cardiovascular pathology (*n* = 9), nephrologic disorders and renal replacement therapy (*n* = 13), pulmonary diseases (*n* = 4), gastrointestinal and hepatopancreatic diseases (*n* = 11), autoimmune and rheumatologic conditions (*n* = 3), neurologic and ophthalmologic conditions (*n* = 2), onco-hematologic disorders (*n* = 11), surgical patients (*n* = 6), traumatology (*n* = 2), and transplanted patients (*n* = 7).

**Figure 2:**
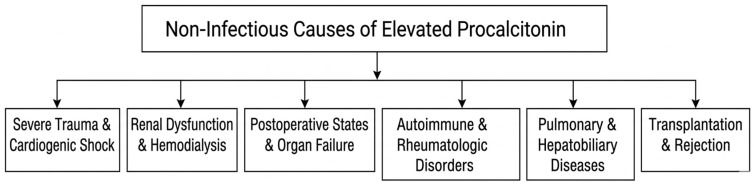
Simplified schematic overview of major non-infectious contexts associated with elevated procalcitonin.

A complete overview of study characteristics (author, year, design, sample size, patient population, PCT results and key findings), as well as detailed numeric PCT values and diagnostic performance metrics is available in the Supplementary Material (Supplementary Tables S1–S11), while the main text focuses on a narrative synthesis of findings by clinical domain. The evidence was grouped into clinically coherent domains; a simplified overview of the major clinical contexts is provided in Figure 2 and guided the narrative synthesis presented below.

### 3.1. Procalcitonin and Systemic Responses

Across diverse non-infectious conditions, PCT demonstrated variable but often clinically relevant elevations. Marked sterile increases were reported in cardiogenic shock, severe trauma, and after cardiopulmonary bypass, in some cases overlapping ranges typically associated with sepsis [16,17,18,19]. Heatstroke induced rapid PCT responses, and higher levels were paradoxically associated with improved survival in the included evidence [20]. In contrast, uncomplicated postoperative states and stable organ-failure cohorts without infection generally showed low PCT values, and low concentrations retained good discriminatory performance for identifying bacterial infection when clinical suspicion was present [21,22].

### 3.2. Procalcitonin and Cardiovascular Disorders

Across cardiovascular and neurological cohorts, PCT levels ranged from low to mildly elevated in the absence of infection, with renal dysfunction, systemic congestion, and inflammatory activation identified as key determinants of variability [23,24,25,26,27,28,29,30,31]. In heart failure, higher PCT showed inconsistent associations with short-term mortality and rehospitalization and offered limited diagnostic or prognostic value in acute dyspnea or coronary artery disease [23,24,25,26,27,28]. In acute ischemic stroke, PCT correlated with stroke severity, lesion burden, and the development of stroke-associated infections, and higher concentrations independently predicted long-term functional decline and mortality [29,30]. In coronary artery disease, a study showed that PCT increased with disease severity, being higher in acute coronary syndrome and correlating with the number of affected arteries [31]. Several studies reported improved prognostic discrimination when PCT was integrated with established clinical scales, indicating heterogeneous yet context-dependent utility across the included populations [23,24,25,26,27,28,29,30,31].

Overall, PCT offered limited standalone diagnostic or prognostic value in stable cardiovascular disease, but may add context-dependent information when interpreted alongside renal status and integrated into established clinical severity frameworks.

### 3.3. Procalcitonin in Renal and Dialysis-Related Disorders

PCT levels increased progressively with worsening renal function, showing mild elevation in non-dialysis CKD and substantially higher baseline values in ESRD patients, particularly those initiating hemodialysis, with changes independent of infection and strongly correlated with reduced GFR and systemic inflammation [32,33,34]. Hemodialysis populations frequently demonstrated elevated baseline PCT despite absence of infection—driven by uremic inflammation and impaired clearance—with higher prevalence in diabetics, elderly patients, and children on HD; PCT typically decreased during dialysis, especially with high-flux membranes, but often remained above normal [35,36,37,38,39,40]. Trimarchi et al. suggested that dialysis-related baseline elevations may extend beyond standard reference ranges and that higher concentrations are more suggestive of infection than modest chronic elevations in stable HD patients [40].

Peritoneal dialysis patients showed moderately elevated PCT compared with controls, though less consistently than HD, and during PD peritonitis PCT rose and declined with therapy, demonstrating high specificity but moderate sensitivity for infection [34,41,42]. In autoimmune nephrological diseases, PCT remained normal in non-infected patients and increased only in bacterial infection, showing high diagnostic accuracy. In renal insufficiency, PCT was normal to mildly elevated except in chronic CAPD patients, while septic patients exhibited marked increases [43]. Among critically ill patients, evidence for PCT as a predictor of AKI was context-dependent; associations were more consistent in non-septic cohorts than in sepsis [44].

Collectively, renal evidence indicates that kidney dysfunction is a major confounder: isolated PCT elevation in CKD/ESRD should not be equated with infection without supportive clinical findings and trend-based assessment.

### 3.4. Procalcitonin in Pulmonary Diseases

Across pulmonary cohorts, PCT concentrations were generally low in non-infectious conditions, indicating limited value for characterizing non-infectious pulmonary pathology as a primary aim [45,46,47,48]. In pleural disease, pleural fluid PCT levels were reported to be low in non-infectious effusions, including transudates, tuberculous pleurisy, and most malignant effusions. By contrast, substantially higher pleural PCT values were observed in purulent pleural collections (e.g., empyema/parapneumonic effusions), supporting the interpretation that marked pleural PCT elevations are more consistent with infectious pleural disease than with non-infectious etiologies [45,46]. In emergency department populations presenting with dyspnea, low PCT values were associated with non-infectious causes (including heart-failure-related dyspnea), whereas higher values were reported in infectious presentations [47,48]. Therefore, in this setting, PCT appears most useful as an adjunct marker to reduce the likelihood of bacterial infection when values are low rather than as a discriminator among non-infectious pulmonary diagnoses [45,46,47,48].

Thus, in pulmonary presentations PCT is most informative as an adjunct to reduce the likelihood of bacterial infection when low, rather than as a classifier of non-infectious pulmonary etiologies.

### 3.5. Procalcitonin in Gastrointestinal and Hepatobiliary Diseases

Overall, in gastrointestinal and hepatobiliary disorders, the evidence indicates that PCT is usually within reference ranges or only mildly elevated in uncomplicated non-infectious disease states, whereas substantial increases tend to occur in the context of severe systemic inflammation, organ dysfunction, or bacterial complications [49,50,51,52,53].

Several studies reported modest sterile PCT elevations in severe hepatitis and decompensated cirrhosis even without documented bacterial infection, which may reduce the specificity of conventional cutoffs in advanced liver disease [49]. This pattern is biologically plausible given systemic inflammatory activation, impaired clearance, and increased gut permeability/endotoxin translocation in cirrhosis; therefore, borderline elevations should be interpreted alongside cultures, imaging, and clinical trajectory rather than as standalone evidence of infection [49,50,51,52].

In liver disease cohorts, PCT generally remained normal in uncomplicated cirrhosis and MASLD, with mild non-infectious elevations described in acute alcoholic or viral hepatitis and in some non-infected cirrhotic patients. Marked increases were reported predominantly in cirrhotic patients with concomitant bacterial infection, and higher PCT levels were associated with adverse outcomes, including mortality, with incremental prognostic value when incorporated into multivariable models [50,51,52]. Clinically, these findings support PCT mainly as an adjunct marker for systemic bacterial complications and for risk stratification in severe liver disease, rather than for etiological classification of hepatic inflammation.

In colitis, PCT showed limited discriminatory performance for differentiating bacterial from nonbacterial etiologies, indicating that both PCT alone and combined with CRP is insufficient for etiologic classification in this setting [53]. However, low PCT may still help reduce the likelihood of systemic bacterial complications in ambiguous presentations, whereas elevations should prompt evaluation for complications rather than labeling colitis etiology.

In acute pancreatitis, several prospective studies reported higher PCT levels in severe disease, particularly when organ failure developed, and early or sustained elevations were described as useful for identifying patients at risk of severe courses [54,55,56,57,58,59]. One prospective study reported that early PCT elevation was associated with subsequent organ failure and appeared to perform favorably compared with conventional severity markers in that cohort [54]. A second prospective cohort suggested that PCT-based strategies could identify high-risk patients early and may provide strong negative predictive value relative to other inflammatory markers, although performance estimates varied by timing and outcome definition [55]. Another study supported the potential utility of a low PCT threshold for early identification of severe pancreatitis, particularly for ruling out severe courses when PCT remained low [56]. However, other cohorts reported poor accuracy for severity stratification, with PCT performing worse than conventional inflammatory markers and showing no consistent differences across etiologic subgroups [58,59]. Taken together, the evidence suggests that serial trends (persistence vs rapid decline) and the broader inflammatory phenotype may be more informative than a single early value for risk stratification [54,55,56,57,58,59].

After endoscopic retrograde cholangiopancreatography (ERCP), PCT increases were generally modest and did not reach levels typically observed in clinically significant bacterial infection, consistent with procedure-related, non-infectious inflammatory activation rather than severe systemic pathology [57].

Overall, gastroenterology evidence supports PCT mainly for identifying systemic bacterial complications and high-inflammatory phenotypes, while mild elevations in advanced liver disease and heterogeneity in pancreatitis limit its value as a standalone discriminator.

### 3.6. Procalcitonin in Autoimmune and Rheumatologic Conditions

Across rheumatologic conditions, PCT generally remained low during non-infectious inflammatory disease. In SLE and AAV, PCT generally remained low during sterile disease activity (even with renal involvement) but increased clearly during systemic bacterial infection, supporting its utility for distinguishing infection from flare [60].

In acute arthritis, serum PCT was significantly higher in bacterial arthritis than in rheumatoid or crystal-induced arthritis, although synovial fluid PCT overlapped between bacterial and crystal-induced forms, limiting discriminatory value [61]. In Kawasaki disease, PCT could be substantially elevated despite non-bacterial etiology and higher levels were associated with coronary involvement, highlighting a setting where sterile inflammation can mimic infectious PCT patterns [62].

Overall, autoimmune evidence supports PCT as a useful adjunct for distinguishing bacterial infection from sterile flare in selected diseases, while acknowledging exceptions where sterile inflammation can markedly elevate PCT.

### 3.7. Procalcitonin in Neurologic and Ophthalmologic Conditions

Serum PCT showed strong discriminatory potential for bacterial meningitis in the included evidence, remaining low in abacterial cases and rising in bacterial infection; persistent elevation was associated with poorer evolution. Given the limited sample size, PCT should be integrated with CSF parameters and microbiology rather than used as a standalone test [63].

In ophthalmology, however, vitreous PCT showed limited discriminatory value: although levels were higher in infectious endophthalmitis than in non-infectious retinal disorders, elevated PCT was also common in non-infectious conditions, and culture-negative endophthalmitis overlapped with controls, resulting in non-significant AUC performance and poor ability to distinguish infectious from non-infectious disease [64].

### 3.8. Procalcitonin in Onco-Hematologic Disorders

Across oncohematologic conditions, PCT demonstrated highly variable reliability. It performed well as a tumor-associated biomarker in medullary thyroid cancer, correlating strongly with calcitonin and reflecting active disease [65]. However, in pediatric oncology and transplant-related inflammation, substantial non-infectious PCT elevations were common: T-cell antibody therapy, alemtuzumab, IL-2, granulocyte transfusions, mucositis, and acute GvHD frequently produced PCT levels overlapping with sepsis due to cytokine surges, immune-cell destruction, and endotoxin translocation, limiting diagnostic specificity [66,67,68,69]. In adult hemato-oncology, PCT often outperformed CRP for distinguishing infection from drug-related or tumor fever in the included evidence, although neutropenic FUO could still present with unexpectedly elevated PCT, limiting the reliability of standard exclusion thresholds [70,71]. In lymphoid malignancies, combining PCT with other inflammatory markers (e.g., ratios) improved discrimination between tumor fever and infection in some cohorts [72]. In febrile neutropenic chemotherapy patients, low PCT values supported infection exclusion in some cohorts, whereas pediatric oncology populations showed frequent false-positive elevations that limited diagnostic usefulness [73,74]. Beyond infection diagnostics, PCT rose with tumor burden in liver malignancies, reaching the highest levels in metastatic disease, while IL-6 more specifically reflected hepatic metastatic involvement [75].

Overall, onco-hematology evidence highlights substantial risk of false positives from cytokine-driven inflammation; therefore, low PCT values may be more useful for ruling out infection than elevated values for ruling it in.

### 3.9. Procalcitonin in Surgical Conditions

Across surgical cohorts, postoperative PCT rose predominantly because of non-infectious inflammatory activation, with peak values and kinetics varying by procedure type. In major cardiovascular surgeries, including aortic dissection repair and on-pump cardiac surgery, PCT increased early after surgery and correlated with cardiopulmonary bypass duration, with higher levels consistently associated with acute kidney injury, severe complications, multiple organ dysfunction, and mortality [76,77,78]. Extremely elevated concentrations in high-risk cardiac surgery patients occurred in both infectious and non-infectious complications, reflecting CPB-induced SIRS rather than bacterial infection [79]. In non-cardiac procedures, postoperative PCT kinetics reflected surgical trauma, showing lower and rapidly declining values in uncomplicated courses but higher and/or persistent elevations in patients with complications [80]. After pancreatoduodenectomy, PCT normalized over the first postoperative days in uncomplicated cases, whereas complicated and fatal outcomes were characterized by persistent elevations, consistent with sustained inflammatory response and possible endotoxin translocation [81].

Overall, surgical evidence supports trend-based interpretation: early postoperative elevations may be sterile, whereas persistence or secondary rises are more consistent with complications and warrant targeted evaluation for infection and non-infectious causes.

### 3.10. Procalcitonin in Traumatology

In intracerebral hemorrhage, admission PCT elevation showed prognostic relevance in the included evidence, aligning with injury burden and poorer outcomes [82]. Conversely, in febrile ICH cohorts, PCT did not differentiate infectious from non-infectious etiologies; non-infectious fever frequently resulted from neurogenic hyperthermia, drug reactions, venous thromboembolism, or transfusion-related responses [83]. These findings suggest that, in neurocritical settings, PCT may contribute to early prognostic assessment but has limited utility for discriminating infectious from non-infectious fever without corroborating clinical and microbiological data.

### 3.11. Procalcitonin in Transplanted Patients

Across solid-organ transplantation, PCT remained low during non-infectious states—including acute rejection, viral infections, postoperative SIRS, and immunosuppression—while rising markedly in systemic bacterial or fungal infections. In heart, lung, and heart–lung transplantation, rejection and viral syndromes generally showed low PCT values comparable to baseline, whereas systemic infection produced clearer elevations, supporting PCT as an adjunct marker when infection versus rejection is clinically uncertain [84,85]. In liver and pancreas transplantation, postoperative trauma caused transient PCT increases, but acute rejection did not elevate PCT, enabling reliable discrimination from infection; context-adapted thresholds and persistent elevation patterns were reported to improve discrimination between infection and non-infectious causes and to align with worse outcomes in complicated courses [86,87,88]. Major postoperative complications were associated with disproportionately elevated PCT, and hepatic vein measurements suggested intrahepatic production during non-infectious injury [89]. Elevated donor PCT also independently predicted early graft-failure mortality after heart donation, outperforming CRP and reflecting underlying donor organ dysfunction [90]. Overall, transplantation evidence supports PCT as a useful adjunct to distinguish systemic bacterial infection from rejection/viral syndromes, while recognizing that early postoperative inflammation and major complications can transiently elevate PCT.

## 4. Discussion

Across the included studies, non-infectious conditions associated with increased PCT encompassed severe tissue injury (e.g., trauma and major surgery), shock states, acute pancreatitis, extensive burns, malignancies (including neuroendocrine tumors), autoimmune/inflammatory disorders, and organ dysfunction syndromes [91,92]. The magnitude and kinetics of PCT elevation varied widely between conditions and across patient subgroups, reflecting differences in the intensity of the insult, the host inflammatory and metabolic response, and the timing of sampling relative to symptom onset or the triggering event [92,93]. Importantly, PCT concentrations reported in sterile inflammatory settings frequently overlapped with values observed in confirmed bacterial infection, creating a clinically relevant risk of diagnostic misclassification when PCT is interpreted in isolation [91,92,93].

Thus, PCT should be understood as a marker that can reflect the burden of systemic inflammation and tissue injury rather than a pathogen-specific signal [14,92]. In high-acuity settings—particularly postoperative, trauma, burn, shock, and pancreatitis cohorts—PCT elevations were often described in the absence of microbiologically confirmed infection, and several studies reported that values could reach ranges traditionally considered suggestive of bacterial sepsis [91,92,93]. This overlap has practical consequences: reliance on static numerical cutoffs without careful consideration of clinical context may promote unnecessary antibiotic exposure, trigger inappropriate escalation of care, or contribute to mislabeling sterile inflammation as sepsis [14,94]. Conversely, dismissing elevated PCT as “always non-specific” may delay recognition of true infection in patients who are simultaneously exposed to strong non-infectious inflammatory stimuli. The evidence therefore supports a context- and trajectory-based approach, prioritizing patterns over single values [95,96].

Mechanistically, research supports two broad pathways for non-infectious PCT elevation. The first is cytokine-driven systemic inflammation induced by major tissue injury, ischemia, hypoxia, and necrosis. In trauma, postoperative stress, circulatory shock, burns, and acute pancreatitis, activation of the innate immune response and release of pro-inflammatory mediators are believed to trigger extrathyroidal PCT expression in multiple parenchymal tissues. In this model, PCT behaves as an acute-phase reactant that can rise rapidly and may track with overall inflammatory burden and the development of organ dysfunction. The second pathway is tumor-associated production, most consistently described in medullary thyroid carcinoma and other neuroendocrine malignancies capable of producing calcitonin gene-related peptides. In such cases, persistently elevated or progressively rising PCT can occur without the clinical phenotype of systemic inflammation, and interpretation as infection may be misleading if malignancy-associated secretion is not considered [97,98].

These mechanistic frameworks help explain clinically observed patterns across the disease categories synthesized in this review. After major surgery and severe trauma, PCT frequently rose early in relation to the magnitude of tissue injury and systemic stress, with the most informative interpretation derived from the subsequent course: declining values were generally consistent with recovery from sterile inflammation, while persistent elevation or secondary rises were more concerning for complications, including infection or ongoing tissue injury [95,99]. In shock states, PCT elevation can plausibly reflect hypoperfusion-associated inflammatory dysregulation and endothelial activation, which may occur with or without infection. In pancreatitis and extensive burns, the inflammatory burden and risk of organ failure appear to be important determinants of PCT magnitude. In malignancy, especially neuroendocrine tumors, sustained elevation may reflect autonomous production rather than acute inflammatory activation. Autoimmune and other inflammatory disorders contributed additional contexts in which sterile inflammation may elevate PCT, reinforcing that interpretation must be anchored in the clinical phenotype and competing diagnoses [91,92].

The literature on non-infectious PCT elevation is marked by substantial heterogeneity and context-dependent findings. In systemic inflammation, PCT may reach values overlapping with sepsis in cardiogenic shock, trauma, or after cardiopulmonary bypass, yet remain low in uncomplicated postoperative or organ-failure states, challenging uniform diagnostic cutoffs. In cardiovascular disease, associations with prognosis are inconsistent in heart failure but more robust in ischemic stroke, underscoring variable utility across populations. Renal dysfunction further complicates interpretation, as baseline PCT is frequently elevated in hemodialysis patients without infection, prompting proposals for higher, population-specific thresholds. Gastrointestinal data are similarly conflicting: PCT shows limited discriminatory value in colitis and inconsistent accuracy for severity stratification in acute pancreatitis. In onco-hematology, cytokine-mediated inflammation and immunotherapies often produce false-positive elevations, reducing specificity, although low values may retain high negative predictive value in selected cohorts. Even in neurologic and ophthalmologic settings, strong performance in bacterial meningitis contrasts with poor discrimination in endophthalmitis. Collectively, these discrepancies reflect differences in underlying inflammatory burden, organ dysfunction, assay thresholds, and study design, precluding a single universal interpretative framework for PCT outside infectious contexts.

From a clinical perspective, PCT is as an adjunct marker that is interpreted alongside the pre-test probability of infection, the patient’s trajectory, and additional diagnostic data. In contexts characterized by major sterile inflammatory stimuli (e.g., postoperative, trauma, burns, pancreatitis, shock), single-threshold strategies are particularly vulnerable to false-positive interpretation [94,96]. Serial measurement can improve interpretability by distinguishing expected transient elevations related to sterile injury from sustained or rising trajectories that warrant targeted reassessment. Such reassessment should include careful clinical examination, microbiology when appropriate, imaging, evaluation for organ dysfunction, and consideration of alternative non-infectious drivers (e.g., ongoing ischemia/necrosis, uncontrolled sterile inflammation, or malignancy-associated production). In stewardship-oriented practice, PCT should not be used as the sole trigger to initiate antibiotics in these complex scenarios; rather, it should contribute supportive information to an integrated decision process [94,96].

Implications for clinical practice extend beyond a simple differentiation between infectious and non-infectious situations. Several included studies suggested that PCT may also provide prognostic information in selected non-infectious conditions, often correlating with severity, organ dysfunction, and mortality risk. However, prognostic interpretation is highly condition-specific and depends on consistent timing of measurement and clinically meaningful endpoints [99]. Where PCT is used for prognostic enrichment, it should be incorporated into multivariable frameworks rather than interpreted as a single decisive marker.

The recognized limitations of PCT in distinguishing infection from sterile systemic inflammation have motivated the evaluation of complementary sepsis biomarkers with distinct biological targets. Recent evidence highlights neutrophil CD64, soluble triggering receptor expressed on myeloid cells-1 (sTREM-1), and presepsin (soluble CD14 subtype) as promising diagnostic adjuncts that may, in selected settings, improve discrimination beyond conventional inflammatory markers [100,101,102,103,104,105,106]. In parallel, biomarkers reflecting endothelial dysfunction and disease severity—most notably pro-adrenomedullin (MR-proADM)—and markers of chronic immune activation such as soluble urokinase-type plasminogen activator receptor (suPAR) have emerged as particularly informative for prognostic stratification, potentially adding value when PCT kinetics are difficult to interpret [107,108,109,110,111,112,113]. Importantly, several of these candidates share clinically relevant confounders with PCT, as presepsin is influenced by renal dysfunction, suPAR may be elevated in cardiovascular disease, malignancy, and autoimmune conditions, and MR-proADM can increase with organ dysfunction regardless of etiology [114,115].

Therefore, in the clinical scenarios emphasized in this review—postoperative inflammation, major trauma, renal replacement therapy, and other non-infectious inflammatory states—novel biomarkers should be viewed as complementary rather than definitive tests, with maximal utility when interpreted longitudinally and integrated with microbiology, imaging, and clinical trajectory. Finally, multi-biomarker panels and data-driven algorithms that combine markers capturing complementary pathophysiology (e.g., innate immune activation, endothelial injury, and organ dysfunction) represent a promising direction to improve discrimination between infectious and non-infectious systemic inflammation and to support antimicrobial stewardship [116,117,118].

Limitations of this review should be considered when applying these conclusions. First, the included studies were heterogeneous with respect to patient populations, clinical definitions, assay platforms, reporting units, and measurement timing. This heterogeneity precluded meta-analysis and limited direct quantitative comparisons across conditions. Second, many cohorts did not apply uniform, gold-standard adjudication for infection, which can bias estimates of overlap between sterile inflammation and bacterial infection. Third, some disease entities—particularly rare malignancies and infrequent autoimmune flares—were represented by small case series, increasing imprecision and susceptibility to selection bias. Fourth, restricting inclusion to English-language publications may have introduced language and publication bias. Finally, inconsistent reporting of PCT kinetics (time from insult, repeated measures, and co-interventions) limited the ability to define condition-specific reference trajectories.

Future research should prioritize standardized reporting of PCT assays, calibration, units, sampling schedules, and clinical definitions to improve comparability and reproducibility. Prospective studies with rigorous infection adjudication and serial sampling are needed to establish condition-specific kinetic patterns and interpretation windows, particularly in high-acuity contexts where sterile inflammation and infection frequently coexist. Comparative studies evaluating PCT in combination with other biomarkers and clinical prediction tools may clarify whether multi-parameter strategies outperform single-marker thresholds in differentiating sterile inflammation from infection and in guiding antimicrobial stewardship. Additional datasets are also required for less common etiologies, including neuroendocrine malignancies and rare autoimmune conditions, to better characterize the prevalence and typical magnitude of non-infectious PCT elevation.

## 5. Conclusions

A wide range of non-infectious conditions—including major trauma, surgery, shock states, acute pancreatitis, extensive burns, malignancies (particularly neuroendocrine tumors), and autoimmune/inflammatory disorders—can be associated with substantial PCT increases through cytokine-driven systemic inflammation and, in selected settings, tumor-associated production. To minimize diagnostic error and unnecessary antimicrobial exposure, PCT should be interpreted within the clinical context and in conjunction with other laboratory and imaging findings, with emphasis on serial trends rather than isolated thresholds. Future research should standardize PCT measurement and reporting, define condition-specific kinetics and interpretation windows, and develop integrated diagnostic and stewardship algorithms that combine PCT with clinical parameters and complementary biomarkers. This systematic review provides a structured framework for interpreting elevated PCT beyond infection and supports more nuanced decision-making in complex inflammatory states.

## Data Availability

Not applicable.

## Supplementary Materials

The following supporting information can be downloaded at: https://www.mdpi.com/article/10.3390/medicina62030464/s1, Supplementary File S1. Full electronic search strategies. Table S1. Studies evaluating PCT in systemic inflammatory responses. Table S2. Studies evaluating PCT in cardiovascular pathology. Table S3. Studies evaluating PCT in nephrologic diseases and renal replacement therapy. Table S4. Studies evaluating PCT in pulmonary diseases. Table S5. Studies evaluating PCT in gastrointestinal and hepatopancreatic diseases. Table S6. Studies evaluating PCT in autoimmune and rheumatologic conditions. Table S7. Studies evaluating PCT in neurologic ophthalmologic conditions. Table S8. Studies evaluating PCT in onco-hematologic disorders. Table S9. Studies evaluating PCT in surgical patients. Table S10. Studies evaluating PCT in traumatology. Table S11. Studies evaluating PCT in transplanted patients

## Abbreviations

The following abbreviations are used in this manuscript:

AAV	ANCA-associated vasculitis
AKI	Acute kidney injury
ARDS	Acute respiratory distress syndrome
AUC	Area under the curve
CAPD	Continuous ambulatory peritoneal dialysis
CKD	Chronic kidney disease
CRP	C-reactive protein
ERCP	Endoscopic retrograde cholangiopancreatography
ESRD	End-stage renal disease
FUO	Fever of unknown origin
GFR	Glomerular filtration rate
HD	Hemodialysis
ICH	Intracerebral hemorrhage
IL-2	Interleukin 2
IL-6	Interleukin 6
JBI	Joanna Briggs Institute
MASLD	Metabolic dysfunction-associated steatotic liver disease
MEDLINE	Medical Literature Analysis and Retrieval System Online
NOS	Newcastle–Ottawa Scale
NPV	Negative predictive value
PCT	Procalcitonin
PD	Peritoneal dialysis
PRISMA	Preferred Reporting Items for Systematic Reviews and Meta-Analyses
SIRS	Systemic inflammatory response syndrome
SLE	Systemic lupus erythematosus
