# Supplementary material for: Non-Infectious Causes for Elevated Procalcitonin
Source: Medicina (Kaunas). 2026 Feb 28;62(3):464. doi: 10.3390/medicina62030464 (PMC13028110; doi:10.3390/medicina62030464)
Supplement: Supplementary file 1 [file medicina-62-00464-s001.zip › medicina-4144928-supplementary.pdf]

Table S1. Studies evaluating PCT in systemic inflammatory responses

Author (Year)	Participants	Study Design	PCT Results	Key Findings
Brunkhorst et al. (1999)	55 with shock:<br>29 cardiogenic shocks<br>26 septic shocks	Retrospective	Baseline:<br>Cardiogenic shock 1.16 ng/mL (0.54)<br>Septic shock 83.03 ng/mL (36.56)<br>Peak:<br>Cardiogenic shock 33.32 ng/mL (11.54)<br>Septic shock 164.48 ng/mL (52.10)<br>Statistically significant difference	Cardiogenic shock without infection caused substantial non-infectious PCT elevation (mean peak 48 ng/mL), correlating with fever severity and cytokine activity despite negative cultures. The rise occurred primarily in patients with prolonged inflammatory complications and was minimal in early deaths or uncomplicated survivors. PCT values overlapped with septic shock, limiting discriminatory utility between severe sepsis and complicated cardiogenic shock.
Wanner et al. (2000)	405 injured	Retrospective	Total: 2.0 ± 0.4 ng/mL<br>Severe injury: 3.3 ± 0.8 ng/mL<br>Cutoff value 1.5ng/mL<br>specificity 77.3%<br>sensitivity 75.6%<br>Statistically significant difference	Severe trauma produced a proportional non-infectious PCT rise, peaking on days 1－3 and reaching up to 38.2 ng/mL in the absence of infection. Patients with ISS ≥25 showed higher levels (mean 3.3 ng/mL), and PCT increased with non-infectious SIRS, though not with SIRS severity. A 1.5 ng/mL cutoff identified early sepsis risk with ~75–77% sensitivity and specificity. Persistent, markedly elevated PCT values were associated with sepsis and severe MODS.
Hensel et al. (1998)	40 undergoing coronary artery bypass surgery:<br>23 No SIRS<br>8 SIRS without ALI<br>9 SIRS with ALI	Prospective	No SIRS 0.9 ± 1.0 ng/mL<br>SIRS without ALI 0.9 ± 0.7 ng/mL<br>SIRS with ALI 8.0 ± 2.7 ng/mL<br>Sensitivity 100%<br>Specificity 100%<br>Statistically significant difference	Severe non-infectious SIRS with acute lung injury after cardiopulmonary bypass produced marked PCT elevations (5–16 ng/mL), whereas patients without SIRS had normal levels. PCT was significantly higher in ALI and strongly correlated with pulmonary dysfunction (r = 0.797). In SIRS patients, it predicted ALI with 100% sensitivity and specificity, outperforming other inflammatory markers and reflecting non-infectious pulmonary inflammation relevant for early postoperative organ dysfunction detection.
Brunkhorst et al. (1999)	27 with ARDS:<br>17 Infectious<br>10 Non-infectious	Prospective	Non-infectious ≤ 2.9 ng/mL<br>Infectious > 5 ng/mL<br>Statistically significant difference	Nonseptic ARDS demonstrated low to moderately elevated PCT levels (≤2.9 ng/mL), with no overlap with septic ARDS, where values were consistently >5 ng/mL.

Nylen et al. (1997)	35 in total:<br>25 with heatstroke<br>10 controls	Prospective	Heatstroke: 3.4 ± 1.0 ng/mL<br>Control: 0.17 ± 0.04 ng/mL<br>Statistically significant difference	Heatstroke caused a rapid, marked non-infectious PCT rise (mean 3.4 ng/mL on admission), with further increases at 6 hours and sustained elevation at 24 hours. Higher admission levels (>10 ng/mL) paradoxically predicted better survival, whereas minimal elevation indicated poor prognosis. PCT was ~20-fold higher than controls and correlated nearly significantly with APACHE II scores, supporting its utility as an early diagnostic and prognostic marker in heatstroke.
Zhao et al. (2014)	142 with SIRS after cardiac surgery:<br>47 infectious<br>95 non-infectious	Retrospective	Non-infectious 0.10 ng/mL (0.06-0.21 ng/mL)<br>Infectious 2.80 ng/mL (1.24-10.20 ng/mL)<br>Cut-off value 0.47 ng/mL<br>AUC_{ROC} = 0.966<br>Sensitivity 91.5%<br>Specificity 93.7%<br>Statistically significant difference	In post-cardiac surgery patients with SIRS, non-infectious cases showed low median PCT levels (0.10 ng/mL), markedly lower than infectious SIRS (2.80 ng/mL). PCT demonstrated excellent diagnostic accuracy (AUC 0.966), outperforming CRP and WBC, with an optimal cutoff of 0.47 ng/mL yielding 91.5% sensitivity and 93.7% specificity for identifying infectious SIRS.
Lee et al. (2022)	420 with positive qSOFA score:<br>141 sepsis<br>137 septic shock<br>142 non-infectious	Prospective	Non-infectious 0.10 ng/mL, 0.05-0.25 ng/mL<br>Sepsis 0.98 ng/mL, 0.35-4.25 ng/mL<br>Septic shock 4.22 ng/mL, 0.88-21.02 ng/mL<br>Cut-off value 0.51 ng/mL<br>AUC_{ROC} = 0.908 (0.877-0.934)<br>Sensitivity 75.5%<br>Specificity 93%<br>Statistically significant difference	Non-infectious organ failure was attributed to diverse etiologies—including CNS, cardiovascular, respiratory, hepatobiliary, renal, and coagulation disorders—and was associated with low PCT levels (median 0.10 ng/mL), significantly below those seen in sepsis (0.98 ng/mL) and septic shock (4.22 ng/mL). PCT showed strong discriminative performance for separating sepsis from non-infectious organ failure (AUC 0.908), outperforming presepsin and CRP, with an optimal cutoff of 0.51 ng/mL yielding 75.5% sensitivity and 93.0% specificity.

Table S2. Studies evaluating PCT in cardiovascular pathology

Author (Year)	Participants	Study Design	PCT Results	Key Findings
Mollar et al. (2014)	261 with acute heart failure	Prospective	0.06 ng/ml (0.04–0.10)	The study reported a low median PCT of 0.06 ng/mL in 261 AHF patients, with eGFR accounting for the largest

				proportion of PCT variability (31.1%). Renal dysfunction, venous congestion, and systemic inflammation—rather than infection—were identified as the primary determinants of PCT levels, with possible contribution from endotoxin translocation.
Boulogne et al. (2017)	75 with heart failure:<br>55 AHF<br>20 CHF (control)	Prospective	AHF 0.14 µg/L (0.09–0.21)<br>CHF 0.13 µg/L (0.10–0.21)<br>No statistically significant difference	Median PCT levels were mildly elevated but remained below infection thresholds in both AHF (0.14 µg/L) and stable CHF (0.13 µg/L), with no significant differences between groups. PCT did not correlate with cardiac biomarkers or functional parameters and showed no prognostic value for mortality or readmission, supporting that mild PCT elevation (0.10–0.50 µg/L) is frequent in HF in the absence of infection.
Canbay et al. (2015)	59 in total:<br>21 outpatients with HF<br>19 inpatients with HF<br>19 health controls	Retrospective	Cut-off value 0.09 ng/mL<br>AUC 0.913 (0.830–0.996)<br>Sensitivity 88.9%<br>Specificity 100%<br>PPV 100%<br>NPV 79.2%<br>Outpatients 0.35 (0.001-1.70) ng/mL<br>Inpatients 1.45 (0.25–5.80) ng/mL<br>Control 0.05 (0.02–0.08) ng/mL<br>Statistically significant difference	PCT levels were significantly higher in HF despite exclusion of infectious cases, with median values of 1.45 ng/mL in inpatients, 0.35 ng/mL in outpatients, and 0.05 ng/mL in controls. PCT cut-offs of 0.09 ng/mL (HF vs controls) and 0.53 ng/mL (decompensated vs stable HF) demonstrated high diagnostic performance (AUC 0.913 and 0.870, respectively), supporting its utility for identifying HF and stratifying decompensation severity.
Travaglino et al. (2014)	441 with acute dyspnea:<br>162 AHF<br>239 No AHF<br>40 AHF + No AHF	Prospective	ED admission AUC 0.65<br>30-day AUC 0.70<br>90-day AUC 0.73<br>Cutoff value 0.09 ng/ml<br>AHF 0.09 ng/ml [0.05-0.19]<br>No AHF 0.09 ng/ml [0.05-0.26]<br>AHF + No AHF 0.13 ng/ml [0.075-0.62]<br>Statistically significant difference	PCT demonstrated moderate accuracy for identifying infection at ED admission (AUC 0.65; optimal cutoff 0.09 ng/mL). Baseline PCT predicted 30-day (AUC 0.70) and 90-day (AUC 0.73) mortality in the overall population and showed stronger prognostic value in patients without AHF (AUC up to 0.74), but not in isolated AHF. While PCT alone did not predict rehospitalization, incorporation of PCT into a multimarker panel enhanced risk stratification for mortality and rehospitalization.
Villanueva et al. (2015)	261 with AHF:<br>96 AHF-rehospitalization	Prospective	0.06 ng/mL (0.04–0.10) on admission.<br>Deceased group 0.073 ng/mL (0.047-0.111)	Median admission PCT was low (0.06 ng/mL) but higher among non-survivors (0.073 vs. 0.051 ng/mL). Higher log-transformed PCT independently predicted all-cause mortality (adjusted HR 1.43) and recurrent rehospitalizations (adjusted

	170 All-cause rehospitalization<br>180 deaths<br>(post 2 years)		Non-deceased 0.051 ng/mL (0.027–0.088)<br>Statistically significant difference	IRR 1.22), with patients in the highest PCT quartile showing a two-fold increase in risk. These associations persisted after adjustment for other inflammatory markers, supporting PCT as a marker of immune activation in AHF once infection is excluded.
Loncar et al. (2015)	168 with acute heart failure	Prospective	Baseline 0.066 ng/mL (0.038–0.121)<br>72h 0.062 ng/mL (0.036–0.112)<br>Discharge 0.043 ng/mL (0.027–0.081)<br>3-month follow-up<br>0.038 ng/mL (0.022–0.059)<br>Statistically significant results	Baseline PCT levels were low (median 0.066 ng/mL) and declined further by discharge and follow-up. Higher admission PCT correlated with greater heart failure severity and independently predicted 90-day mortality or rehospitalization, with serial measurements offering stronger prognostic value than single values. PCT performed comparably to NT-proBNP, and its elevation was attributed to endotoxin translocation rather than infection.
Hug et al. (2011)	50 with ischemic stroke:<br>18 SARTI+<br>32 SARTI-	Retrospective	AUC (SARTI) 0.79 (0.61–0.96)<br>Cutoff value 0.25 ng/mL<br>Day 1:<br>0.06 ng/ml (0.04–0.07) (SARTI-)<br>0.09 ng/ml (0.05–0.17) (SARTI+)<br>0.06 ng/mL (0.05–0.1) (total)<br>Day 4:<br>0.07 ng/ml (0.05–0.09) (SARTI-)<br>0.14 ng/ml (0.08–0.39) (SARTI+)<br>0.08 ng/mL (0.05–0.13) (total)<br>Statistically significant difference	Early PCT levels remained low (median 0.06 ng/mL on day 1; 0.08 ng/mL on day 4), with higher day 4 values observed in those who developed stroke-associated infections (0.14 vs. 0.07 ng/mL). Day 1 PCT was not predictive of infection, and infarct volume was a stronger independent predictor of SARTI than PCT (AUC 0.96 vs. 0.79). Using a 0.25 ng/mL cutoff, day 4 PCT demonstrated high specificity (96%) but low sensitivity (42%) for detecting SARTI.
Wang et al. (2015)	576 total:<br>376 with AIS (64 non-survival & 312 survival)<br>200 controls	Prospective	AUC 0.77 (0.66–0.81)<br>Cutoff value 1.03 ng/ml<br>Sensitivity 72.6 %<br>Specificity 77.5 %<br>AIS 0.86 ng/ml (0.15–1.76)<br>Non-survival 3.12 ng/ml (1.83–4.30)<br>Survival 0.54ng/ml (0.09–1.09)<br>Controls 0.03 ng/ml (0.02–0.04)<br>Statistically significant difference	Median PCT levels were significantly higher than in healthy controls (0.86 vs. 0.03 ng/mL) and correlated with stroke severity and lesion size. Elevated PCT independently predicted poor 1-year functional outcome (OR 2.33) and mortality (OR 3.11), outperforming hs-CRP and age, and improved prognostic accuracy when added to NIHSS (AUC up to 0.94).
Sinning et al.	2,131 with CAD:	Retrospective	Baseline:	Median PCT levels were low but higher in patients who experienced cardiovascular events or death, increasing with

(2011)	1951 without CV events<br>180 with CV events		Without CV events 0.015 ng/mL (0.010/0.023)<br>With CV events 0.016 ng/mL (0.010/0.029)<br>Follow-up:<br>Deceased (n=95) (0.021 ng/mL (0.012/0.036)<br>Statistically significant difference	ACS severity and correlating with coronary disease burden. PCT was associated with male sex, diabetes, obesity, and showed moderate correlation with CRP. Although baseline PCT independently predicted cardiovascular mortality (HR 1.34), this association lost significance after CRP adjustment, and PCT did not independently predict composite cardiovascular events, indicating limited prognostic value beyond CRP.

Table S3. Studies evaluating PCT in nephrologic diseases and renal replacement therapy

Author (Year)	Participants	Study Design	PCT Results	Key Findings
Dahaba et al. (2003)	55 patients with end-stage renal failure	Prospective	Pre-hemodialysis 1.82 ± 0.39 ng/ml<br>Baseline of 1.5 ng/ml<br>1st hemodialysis:<br>PCT M_{TR} 79.2 ± 170.9 ng/min<br>PCT Cl 37.7 ± 20.7 ml/min<br>2nd hemodialysis:<br>PCT M_{TR} 34.3 ± 32 ng/min<br>PCT Cl 48.8 ± 16.6 ml/min<br>3rd hemodialysis:<br>PCT M_{TR} 21.1 ± 31.2 ng/min<br>PCT Cl 45.4 ± 10.6 ml/min<br>Statistically significant difference	Nondialyzed ESRD patients exhibited elevated baseline PCT levels (mean 1.82 ± 0.39 ng/mL), with significant reductions after each hemodialysis session and progressive increases in PCT clearance across sequential sessions, supporting a proposed baseline threshold of ~1.5 ng/mL in nonseptic uremic patients.
Herget-Rosenthal et al. (2005)	414 patients:<br>37 Controls<br>281 CKD (stage I-V)<br>31 PD<br>65 HD	Retrospective	Controls 0.10 ± 0.03 ng/ml<br>CKD:<br>Stage I 0.10 ± 0.01 ng/ml<br>Stage II 0.09 ± 0.02 ng/ml<br>Stage III 0.14 ± 0.07 ng/ml<br>Stage IV 0.14 ± 0.08 ng/ml<br>Stage V 0.29 ± 0.21 ng/ml<br>PD 0.38 ± 0.18 ng/ml<br>HD 0.64 ± 0.34 ng/ml<br>Statistically significant difference	PCT levels increased progressively with declining renal function, showing a strong inverse correlation with GFR (r = –0.65), and were highest in CKD stage V and patients undergoing dialysis. PCT correlated with inflammatory markers, including CRP (r = 0.73), and reflected uremia-associated low-grade inflammation.

Lavín-Gómez et al. (2011)	139 patients:<br>69 controls<br>70 end-stage CKD:<br>25 non-dialysis<br>22 peritoneal dialysis<br>23 hemodialysis	Retrospective	Control 0.03 (0.02 to 0.03) ng/ml<br>Non-dialysis 0.12 (0.09 to 0.16) ng/ml<br>Peritoneal dialysis 0.32 (0.20 to 0.46) ng/ml<br>Hemodialysis 0.79 (0.45 to 0.99) ng/ml<br>Statistically significant difference	The team reported progressively higher PCT levels with advancing CKD and initiation of renal replacement therapy, with the highest concentrations observed in hemodialysis patients (0.79 ng/mL) and lower levels in non-dialysis CKD (0.12 ng/mL) and peritoneal dialysis (0.32 ng/mL).
Schmidt et al. (2000)	46 recipients:<br>10 CAPD<br>36 chronic HD	Prospective	Cutoff level <1.5 ng/ml<br>CAPD:<br>0.8 ng/ml (1 out of 10)<br>0.23 ± 0.11 ng/ml (remaining 9)<br>Chronic HD:<br>0.58 ± 0.35 ng/ml (baseline)<br>0.44 ± 0.32 ng/ml (end of HD)<br>Statistically significant difference	Chronic hemodialysis patients frequently exhibited elevated baseline PCT levels (>0.5 ng/mL in 44% of cases), with levels decreasing significantly during dialysis, while CAPD patients generally showed low PCT concentrations. The authors proposed a higher diagnostic cutoff (>1.5 ng/mL) for infection in chronic HD patients.
Level et al. (2001)	62 on maintenance hemodialysis:	Prospective	0.69 ± 0.81 ng/ml (total population)<br>Infectious 1.15 ±1.5 ng/ml (18%)<br>Non-infectious 0.58±0.38 ng/ml<br>Statistically significant difference	In 62 chronic hemodialysis patients, 57% exhibited elevated baseline PCT levels (>0.5 ng/mL) despite a low prevalence of infection (18%), and infected patients showed significantly higher PCT concentrations than non-infected individuals. PCT correlated positively with CRP, IL-6, and neopterin, reflecting an inflammatory rather than infectious or nutritional signal.
Visvardis et al. (2005)	120 on maintenance hemodialysis	Prospective	> 0.5 ng/ml in 38% of total population (74% diabetics and 39.78% elderly)	38% of maintenance hemodialysis patients had elevated PCT levels (>0.5 ng/mL), with higher prevalence in diabetics (74%) and elderly patients (≈40%). PCT correlated strongly with CRP and IL-6, supporting its association with chronic inflammation in this population.
Lorton et al. (2007)	8 children on hemodialysis<br>(24 samples):	Prospective	0.75 ± 0.07 ng/ml (15 out of 21 samples pre-hemodialysis)<br>0.44±0.04 ng/ml (reduction of 40% after hemodialysis)	Elevated baseline PCT levels in children undergoing chronic hemodialysis (mean 0.75 ng/mL), with most samples exceeding 0.5 ng/mL, and observed a 40% reduction

	21 non-infectious samples<br>3 infectious samples		Statistically significant difference	following dialysis, although post-dialysis levels frequently remained elevated. PCT showed a modest positive correlation with CRP.
Montagnana et al. (2009)	44 on hemodialysis:<br>22 high-flux<br>22 low-flux	Prospective	High-flux group:<br>0.50 ng/ml pre-hemodialysis<br>0.26 ng/ml post-hemodialysis<br>Statistically significant difference<br>Low-flux group:<br>0.41ng/ml pre-hemodialysis<br>0.42ng/ml post-hemodialysis<br>Not statistically significant difference	Elevated baseline PCT levels in all non-infected hemodialysis patients, with significant post-dialysis reductions observed only in those treated with high-flux membranes, while low-flux membranes produced no meaningful change in PCT concentrations.
Opatrná et al. (2005)	56 total:<br>28 on PD<br>28 controls	Prospective	Peritoneal dialysis patients:<br>0.33(0.17-1.81) ng/ml<br>Controls:<br>0.18(0.14-0.33) ng/ml<br>Statistically significant difference	Peritoneal dialysis patients exhibited moderately elevated plasma PCT levels (0.33 ng/mL) compared with healthy controls (0.18 ng/mL), with PCT correlating positively with CRP and inversely with renal clearance markers. PCT was detectable in dialysate and strongly correlated with plasma concentrations, indicating transperitoneal movement rather than peritoneal generation.
Guz et al. (2006)	159 total:<br>51 on PD<br>74 on HD<br>34 controls	Prospective	AUC_{ROC} 0.80 (0.656–0.949)<br>PD patients:<br>>0.5 ng/ml in 5.8%<br>0.2 ng/ml (0.1-0.3)<br>HD patients:<br>>0.5 ng/ml in 17.5%<br>>1.5 ng/ml in 4.0%<br>Control:<br>Normal values (<0.5 ng/ml)<br>Statistically significant difference	Mildly elevated baseline PCT levels in both PD and HD patients compared with controls, with elevated PCT (>0.5 ng/mL) occurring in 5.8% of PD and 17.5% of HD patients without infection. In PD peritonitis episodes, PCT increased to a median of 1.0 ng/mL and declined after antibiotic therapy, demonstrating a sensitivity of 62% and specificity of 94% for peritonitis diagnosis.
Steinbach et al. (2004)	125 total:<br>23 Group 1 (CRF)<br>20 Group 2 (CAPD)<br>20 Group (HD <1)	Prospective	Group 1: 0.1 µg/L (0-0.2)<br>Group 2: 1.1 µg/L (0.1-11.5)<br>Group 3: 0.3 µg/L (0-270.5)<br>Group 4: 0.6 µg/L (0.1-6.1)	The study reported normal or only mildly elevated PCT levels in chronic renal failure and hemodialysis patients, whereas non-infected CAPD patients demonstrated higher

	22 Group 4 (HD >1)<br>20 Group 5a (sepsis with ARF)<br>20 Group 5b (sepsis without ARF)		Group 5a: 9.9 µg/L (1.3-245)<br>Group 5b: 35 µg/L (7-231)<br>Specificity 94%<br>Sensitivity 94%<br>Statistically significant difference	concentrations (1.1 µg/L). In cases of sepsis, PCT increased markedly (median 9.9–35 µg/L). PCT showed high diagnostic accuracy for bacterial infection (94% sensitivity and specificity), with a proposed infection cutoff >1.5 µg/L for hemodialysis patients.
Jeeha et al. (2018)	201 patients admitted to ICU:<br>74 developed AKI (36.8%):<br>36 stage 1<br>19 stage 2<br>19 stage 3<br>127 did not develop AKI	Retrospective	< 10 ng/ml 28 (23.9%) developed AKI<br>≥10 ng/ml, 46 (54.8%) developed AKI<br>Statistically significant difference<br>(for ≥10 ng/ml):<br>Sensitivity 46.2%<br>Specificity 86.5%<br>PPV 50%<br>NPV 84.6%	PCT was not associated with AKI in septic patients, whereas in non-septic critically ill adults, admission PCT ≥10 ng/mL was independently associated with a higher risk of AKI. A cutoff of ≥10 ng/mL demonstrated moderate diagnostic performance for AKI prediction (46.2% sensitivity, 86.5% specificity).
Trimarchi et al. (2013)	84 in total:<br>48 with chronic HD without active infection<br>36 controls	Retrospective	Chronic HD 0.26 ng/mL (0.071-1.14)<br>Controls 0.034 ng/mL (0.02-0.08)<br>Statistically significant difference	Chronic hemodialysis patients without infection showed significantly elevated baseline PCT levels (median 0.26 ng/mL) compared with controls (0.034 ng/mL), with a proposed non-infectious upper threshold of 0.824 ng/mL. Values ≤0.8 ng/mL reflected low-grade sterile inflammation, while levels >2 ng/mL indicated a higher likelihood of infection

Table S4. Studies evaluating PCT in pulmonary diseases

Author (Year)	Participants	Study Design	PCT Results	Key Findings
Wang et al. (2011)	76 with pleural effusion:<br>27 Malignant pleural effusion<br>26 Para-pneumonic effusion<br>8 Transudative pleural effusion<br>8 Tuberculous pleurisy<br>7 Empyema	Prospective	Cutoff value 0.18 ng/ml<br>AUC_{ROC} = 0.776 (with 69.7% sensitivity and 72.1% specificity)<br>Malignant pleural effusion 0.241±0.071 ng/mL<br>Para-pneumonic effusion 1.091±0.355 ng/mL	Pleural fluid PCT levels were low in transudates and tuberculous pleurisy and slightly higher in malignant effusions, while markedly elevated values were observed in infectious effusions such as empyema and parapneumonic effusion. A pleural fluid PCT cutoff of 0.18 ng/mL differentiated empyema/parapneumonic effusions from non-parapneumonic causes with

			Transudative pleural effusion 0.188±0.077 ng/mL<br>Tuberculous pleurisy 0.130±0.069 ng/mL<br>Empyema 5.147±3.056 ng/mL<br>Statistically significant difference	moderate diagnostic accuracy (AUC 0.776; sensitivity 69.7%; specificity 72.1%).
Watanabe et al. (2018)	132 with pleural effusion:<br>94 non-infectious<br>38 infectious	Retrospective	Optimal cut-off point ≥0.11ng/mL<br>AUC_{ROC} = 0.696<br>Senitivity 63.2%<br>Specificity 74.5%<br>Statistically significant difference	Pleural fluid PCT levels were significantly higher in parapneumonic effusions than in tuberculous, malignant, para-malignant, or transudative effusions. A pleural PCT cutoff of ≥0.11 ng/mL distinguished infectious from non-infectious effusions with moderate performance (AUC 0.696; sensitivity 63.2%; specificity 74.5%).
Cinar et al. (2012)	154 with dyspnea:<br>65 CHF exacerbation<br>51 Infectious<br>38 Other	Prospective	0.08 ng/mL (0.05-0.17)<br>Cut-off value 0.25 ng/mL<br>Sensitivity 48.8%<br>Specificity 96.5%<br>Statistically significant difference	A PCT cutoff of 0.25 ng/mL showed very high specificity (96.5%) but moderate sensitivity (48.8%) for identifying infectious causes of dyspnea. The high positive likelihood ratio (13.8) indicated that elevated PCT strongly favored infection, while low values helped exclude non-infectious etiologies such as CHF. Incorporating PCT into clinical evaluation significantly improved diagnostic accuracy for both infectious and non-infectious dyspnea, reaching 90.9% and 94.1%, respectively.
Alba et al. (2016)	453 with dyspnea:<br>30 Pneumonia<br>212 HF without pneumonia<br>30 with both	Prospective	Pneumonia 0.38 ng/mL (0.12-1.40)<br>Without pneumonia 0.06 ng/mL (0.04-0.09)<br>HF and pneumonia 0.62 ng/mL (0.28-3.20)<br>Cutoff value 0.10 ng/mL<br>AUC 0.84<br>Specificity 85%<br>Sensitivity 95%<br>NPV 99%	PCT levels were significantly higher in pneumonia (median 0.38 ng/mL) than in non-pneumonic causes (0.06 ng/mL), with the highest values in patients with both heart failure and pneumonia (0.62 ng/mL). A 0.10 ng/mL cutoff identified pneumonia with high accuracy, especially in suspected HF (95% sensitivity; 99% NPV). Elevated PCT independently predicted 90-day and 1-year mortality and improved prognostic models when combined with natriuretic peptides.

			Statistically significant difference	

Table S5. Studies evaluating PCT in gastrointestinal and hepatopancreatic diseases

Author (Year)	Participants	Study Design	PCT Results	Key Findings
Elefsiniotis et al. (2006)	106 with liver diseases:<br>15 with acute alcoholic hepatitis on cirrhosis (group A)<br>20 with alcoholic cirrhosis without hepatitis and/or bacterial infection (group B)<br>16 with decompensated cirrhosis with infection (group C)<br>42 with uncomplicated viral hepatitis-related cirrhosis (group D)<br>13 with acute icteric viral hepatitis (group E)	Prospective	Group A 0.40 ± 0.30 ng/mL<br>Group B 0.23±0.16 ng/mL<br>Group C 9.80±16.80 ng/mL<br>Group D 0.21±0.14 ng/mL<br>Group E 0.37±0.22 ng/mL<br>Cutoff value 0.75 ng/mL<br>Sensitivity 61%<br>Specificity 99%<br>Statistically significant difference	PCT levels remained normal in uncomplicated cirrhosis, while cirrhotic patients with bacterial infection showed markedly elevated values (mean 9.8 ng/mL). Mild non-infectious PCT increases above 0.5 ng/mL were observed in acute alcoholic hepatitis and acute icteric viral hepatitis despite absence of infection. A higher diagnostic cutoff (0.75 ng/mL) improved specificity for detecting bacterial infection in severe liver disease.
Oruc et al. (2009)	100 in total:<br>50 with NAFLD (steatohepatitis n=20, diffuse steatosis n=27, focal fatty liver n=3)<br>50 controls	Retrospectively	Steatohepatitis 0.06 ng/mL ± 0.01<br>Diffuse steatosis 0.04 ng/mL ± 0.01<br>Focal fatty liver 0.03 ng/mL ± 0.01	Serum PCT levels remained within normal ranges across NAFLD subgroups (≈0.04–0.06 ng/mL) and did not correlate with liver function, BMI, or insulin resistance. In contrast, CRP was significantly elevated in NAFLD, indicating chronic inflammation. The study shows that PCT remains normal in

			Controls 0.06 ng/mL ± 0.01<br>No statistically significant difference	NAFLD and lacks diagnostic utility for this non-infectious liver condition.
Bota et al. (2005)	864 in ICU:<br>79 with cirrhosis:<br>Infected n = 39:<br>Child class A, n = 15<br>Child class B, n = 14<br>Child class C, n = 10<br>Non-infected n = 40:<br>Child class A, n = 13<br>Child class B, n = 13<br>Child class C, n = 14<br>785 no cirrhosis	Prospective	No cirrhosis<br>0.5 ng/mL ± 0.3<br>Cirrhosis (total)<br>0.6 ng/mL ± 0.4<br>Cirrhosis with infection:<br>Child class A 1.2 ng/mL ± 0.8<br>Child class B 1.3 ng/mL ± 0.8<br>Child class C 1.1 ng/mL ± 0.7<br>Cirrhosis without infection:<br>Child class A 0.3 ng/mL ± 0.2<br>Child class B 0.4 ng/mL ± 0.3<br>Child class C 0.4 ng/mL ± 0.3<br>No statistically significant difference	Neither cirrhosis nor Child-Pugh severity significantly altered PCT or CRP levels, with both initial and peak PCT values comparable between cirrhotic and non-cirrhotic groups. Among 40 non-infected cirrhotic patients, only mild non-infectious PCT elevation was observed (mean 0.3 ng/mL). These findings indicate that liver dysfunction has minimal impact on circulating PCT, supporting substantial extrahepatic PCT production.
Chirapongsathorn et al. (2018)	71 with cirrhosis:<br>18 deceased<br>53 non-deceased	Prospective	0.381 ng/mL (0.031-48.7), (total)<br>Deceased 2.62 ng/mL (0.12-48.7)<br>Non-deceased 0.37 ng/mL (0.043-15.98)<br>Cut-off value ≥ 2.62 ng/mL<br>AUC 0.72	Median PCT at admission was 0.381 ng/mL, with non-survivors exhibiting markedly higher levels (2.62 vs. 0.37 ng/mL). A PCT threshold ≥2.62 ng/mL independently predicted 90-day mortality (c-statistic 0.72). Combining PCT with MELD improved prognostic performance (c-statistic 0.84 vs. 0.81 for MELD alone), and the addition of CRP further enhanced discrimination (c-statistic 0.85).

			Sensitivity 53.33%<br>Specificity 91.49%<br>Statistically significant difference	
Lee et al. (2023)	636 patients with colitis:<br>186 bacterial<br>450 non-bacterial	Retrospective	Bacterial 8.46 ± 21.70 ng/mL<br>Non-bacterial 7.98 ± 22.15 ng/mL AUC 0.557 (0.509-0.605)<br>Cutoff value 0.52 ng/mL<br>Sensitivity 54.8%<br>Specificity 54.6%<br>No statistically significant difference	Mean serum PCT levels did not differ between bacterial and nonbacterial cases (8.46 ± 21.70 vs. 7.98 ± 22.15 ng/mL). Diagnostic accuracy was poor (AUC 0.557), and at a 0.52 ng/mL cutoff, PCT showed low sensitivity (54.8%) and specificity (54.6%). Combining PCT with CRP did not enhance diagnostic performance for distinguishing bacterial from nonbacterial colitis.
Kylänpää-Bäck et al. (2001)	57 with acute pancreatitis:<br>30 mild (SEV0)<br>27 severe:<br>11 without organ failure (SEV1)<br>16 Acute respiratory and 9 renal failure (SEV2)	Prospective	T_0:<br>SEV0 0.3 ng/mL (0.1-3)<br>SEV1 0.4 ng/mL (0.2-2.3)<br>SEV2 0.8 ng/mL (0.2-73.5)<br>T_{12}:<br>SEV0 0.3 ng/mL (0.1-1.7)<br>SEV1 0.4 ng/mL (0.3-2.8)<br>SEV2 2.2 ng/mL (0.2-86.6)<br>T_{24}:<br>SEV0 0.3 ng/mL (0.1-1.2)<br>SEV1 0.5 ng/mL (0.3-4)	Admission PCT levels were higher in severe disease compared with mild AP (median 0.3 ng/mL in SEV0, 0.4 ng/mL in SEV1, and 0.8 ng/mL in SEV2). Persistent elevation at 12–24 hours was most pronounced in SEV2 (median 2.2 ng/mL), reflecting intense non-infectious systemic inflammation preceding organ failure. A 24-hour PCT >0.4 ng/mL predicted organ failure with high accuracy (94% sensitivity, 73% specificity), outperforming APACHE II and CRP, and higher PCT values were associated with more rapid progression to organ failure.

			SEV2 2.2 ng/mL (0.4-73.3)<br>Sensitivity 94%<br>Specificity 73%<br>Statistically significant difference	
Kylänpää-Bäck et al. (2000)	162 with acute pancreatitis:<br>38 severe<br>124 mild	Prospective	Day 0:<br>Sensitivity 71%<br>Specificity 84%<br>PPV 52%<br>NPV%<br>Day1:<br>Sensitivity 92%<br>Specificity 84%<br>PPV 53%<br>NPV 97%<br>Statistically significant difference	PCT levels were significantly higher in severe versus mild AP at both admission and 24 hours. PCT detected all cases of organ failure with high sensitivity (86% at admission; 95% at 24 hours) and achieved 100% sensitivity for fatal outcomes when using the higher of the two measurements. While PPVs were similar to CRP and APACHE II, PCT demonstrated superior NPVs. The findings support early PCT as an effective screening marker for identifying severe AP and predicting organ failure.
Bülbüller et al. (2006)	65 with acute pancreatitis:<br>46 mild<br>19 severe	Prospective	Mild 0.34 ng/mL ± 0.27<br>Severe 2.93 ng/mL ±3.2<br>Cutoff 0.5 ng/mL<br>Sensitivity 100%<br>Specificity 84%<br>PPV 73%<br>NPV 100%<br>Statistically significant difference	Admission PCT levels were significantly higher in severe versus mild disease (mean 2.93 vs. 0.34 ng/mL). Mild AP remained largely below 0.5 ng/mL, whereas severe AP consistently exceeded this threshold during the first six days. A cutoff of 0.5 ng/mL identified severe AP with 100% sensitivity, 84% specificity, 100% NPV, and 86% overall accuracy. PCT outperformed Ranson, APACHE II, and CRP for early severity assessment, with decreasing levels reflecting clinical recovery.
Oezcueruemez-Porsch et al. (1998)	94 undergoing ERCP:<br>12 post-ERCP pancreatitis	Prospective	Healthy < 0.08 µg/liter<br>Baseline:<br>Post-ERCP pancreatitis 0.09 ± 0.01µg/liter	PCT increased modestly 24 hours after the procedure from a mean baseline of 0.09 µg/L, with no values exceeding 0.5 µg/L. PCT showed moderate, non-significant correlations with procedural factors such as endoscopy duration, pain score, and cannulation attempts, reflecting a mild non-infectious systemic response consistent with clinically mild post-ERCP

			Peak(24h):<br>Post-ERCP pancreatitis 0.16 ± 0.02μg/liter<br>Statistically significant difference	pancreatitis. PCT rose later and correlated less strongly with procedural trauma than IL-1RA and IL-6.
Pezzilli et al. (2000)	62 total:<br>31 controls<br>31 with acute pancreatitis:<br>19 mild<br>12 severe	Prospective	Control 0.048 - 0.760 ng/ml<br>Cutoff value 0.252 - 0.255 ng/mL<br>AUC 0.456 ± 0.036<br>Sensitivity 21.7%<br>Specificity 83.2%<br>No statistically significant difference	PCT was elevated in AP but did not differentiate mild from severe disease. The optimal severity cutoff (≈0.25 ng/mL) showed poor diagnostic performance (AUC 0.456; sensitivity 21.7%; specificity 83.2%), markedly inferior to SAA and CRP. PCT levels were unaffected by AP etiology.
Melzi D'Eril et al.(2000)	31 with AP:<br>25 biliary origin<br>1 alcohol-induced<br>5 unknown etiology	Prospective	Cutoff value 0.252 - 0.255 μg/L<br>AUC 0.456 ± 0.036<br>$Sensitivity_{MAX}$ 33.3%<br>$Specificity_{MAX}$ 89.5%<br>Not statistically significant difference	PCT was elevated compared with healthy controls but did not significantly differ between mild and severe AP. Severity discrimination was poor (AUC 0.456) with low sensitivity (≤33.3%) despite relatively higher specificity, performing markedly worse than CRP (AUC 0.776). PCT levels were similar across biliary and non-biliary etiologies, supporting its role as a specific marker for bacterial infection rather than for assessing non-infectious AP severity.

Table S6. Studies evaluating PCT in autoimmune and rheumatologic conditions

Author (Year)	Participants	Study Design	PCT Results	Key Findings

Eberhard et al. (1997)	53 with autoimmune disease (397 samples):<br>18 with SLE (226 samples)<br>35 with AAV (171 samples)<br>11 infectious	Retrospective	Non-infectious SLE and AAV: <0.5ng/mL<br>Infectious AAV:<br>1.93 ± 1.19 ng/mL (acute phase)<br>0.61 ± 0.62 ng/mL (antibiotic therapy)<br>Sensitivity 100%<br>Specificity 84%<br>Statistically significant difference	PCT remained low (≤0.5 ng/mL) during active non-infectious disease and showed no association with disease activity or renal dysfunction. In contrast, PCT rose markedly during systemic bacterial infections in AAV patients (mean 1.93 ng/mL), normalizing with treatment and demonstrating high diagnostic performance for infection (100% sensitivity; 84% specificity).
Martinot et al. (2006)	42 with acute arthritis:<br>11 bacterial arthritis<br>18 rheumatoid arthritis<br>13 crystal induced arthritis	Prospective	Serum:<br>Bacterial arthritis (BA) 0.7 ng/mL (<0.08-223.6)<br>Crystal arthritis (CA) <0.08 ng/mL (<0.08-0.69)<br>Rheumatoid arthritis (RA) <0.08 ng/mL (<0.08-0. 61)<br>Synovial (SF):<br>Bacterial arthritis 0.22 ng/mL (<0.08-1.31)<br>Crystal arthritis 0.09 ng/mL (<0.08-0.88)<br>Rheumatoid arthritis <0.08 ng/mL (<0.08-0. 6)<br>55% sensitivity<br>94% specificity<br>Statistically significant difference with RA<br>No statistically significant difference with CA	Serum PCT was significantly higher in bacterial arthritis (median 0.7 ng/mL) than in rheumatoid or crystal-induced arthritis (both <0.08 ng/mL). Synovial fluid PCT was lower in RA than in bacterial arthritis, but values in bacterial and crystal-induced arthritis overlapped, limiting its ability to distinguish between these two conditions.
Okada et al. (2004)	87 total:<br>25 KD<br>17 infectious (bacterial)<br>17 infectious (viral)<br>10 autoimmune<br>18 controls	Prospective	Controls 0.2 ± 0.1 ng/mL<br>KD 2.3 ± 3.0 ng/mL<br>Bacterial infection 2.2 ± 2.9 ng/mL<br>Viral infection 0.4 ± 0.3 ng/mL<br>Autoimmune 0.4 ± 0.4 ng/mL<br>Cutoff value 3 ng/mL<br>Sensitivity 100%<br>Specificity 90%<br>Statistically significant difference	Serum PCT was markedly elevated in acute KD (mean 2.3 ng/mL), at levels comparable to bacterial infections (2.2 ng/mL) and significantly higher than autoimmune and viral conditions (both ~0.4 ng/mL) as well as healthy controls (0.2 ng/mL). PCT effectively distinguished KD from autoimmune diseases when standard inflammatory markers did not, and a cutoff of 3 ng/mL predicted coronary aneurysm formation with high accuracy (100% sensitivity; 90% specificity). Elevated

				PCT in KD reflects intense non-infectious vasculitic inflammation that can mimic bacterial sepsis.

Table S7. Studies evaluating PCT in neurologic ophthalmologic conditions

Author (Year)	Participants	Study Design	PCT Results	Key Findings
Schwarz et al. (2000)	30 with meningitis:<br>16 acute bacterial<br>14 abacterial	Prospective	Abacterial 0.24 ng/mL (0.12-0.29)<br>Bacterial 1.75 ng/mL (0.16-59.92)<br>Cutoff value > 0.5ng/mL<br>Specificity 100%<br>Sensitivity 69%<br>Statistically significant difference	Serum PCT effectively distinguished bacterial from abacterial meningitis, remaining normal and stable over 48 hours in abacterial cases (median 0.24 ng/mL) and markedly elevated in bacterial disease (median 1.75 ng/mL). A cutoff >0.5 ng/mL provided 100% specificity for bacterial etiology, and persistently high or rising values were associated with worse clinical outcomes.
Naik et al. (2021)	74 patients:<br>55 infectious endophthalmitis<br>19 non-infectious (control group)	Prospective	Infectious 117.74 ± 18.97 pg/mL<br>Non-infectious 75.74 ± 26.8 pg/mL<br>Statistically significant difference	Vitreous PCT levels were significantly higher in the infectious group (117.74 ± 18.97 pg/mL vs. 75.74 ± 26.8 pg/mL). However, culture-negative endophthalmitis showed PCT levels comparable to controls, and elevated vitreous PCT was also frequently detected in non-infectious conditions. As a result, diagnostic accuracy was limited, with the AUC not reaching statistical significance due to overlap between infectious and non-infectious groups.

Table S8. Studies evaluating PCT in onco-hematologic disorders

Author (Year)	Participants	Study Design	PCT Results	Key Findings
Woliński et al. (2017)	44 with MTC:<br>20 active diseases<br>24 in remission	Retrospective	Cutoff value 0.95 ng/mL<br>Sensitivity 100.0%<br>Specificity 95.8%<br>Active diseases: 23.45 ng/mL (0.96 - 400)	PCT was a highly accurate marker of active MTC, correlating strongly with calcitonin (r = 0.93). Active disease showed markedly elevated PCT (median 23.45 ng/mL) compared with remission (0.04 ng/mL). A 0.95 ng/mL cutoff identified active MTC with 100% sensitivity and up to 95.8% specificity, outperforming CEA and chromogranin A. Given

			Remission: 0.04 ng/dL (0.02 - 1.0)<br>Statistically significant difference	its analytical stability, availability, and rapid turnaround, PCT was supported as a practical adjunct biomarker for MTC monitoring and imaging guidance.
Dornbusch et al. (2008)	130 patients:<br>110 non-infectious febrile (group A-E)<br>20 Gram-negative sepsis (group F)	Prospective	Group A: 4.34 ng/ml (0.22–176.11)<br>Group B: 10.14 ng/ml (2.73–60.91)<br>Group C: 1.11 ng/ml (0.2–20.2)<br>Group D: 1.43 ng/ml (0.87–4.29)<br>Group E: 0.96 ng/ml (0.34–6.95)<br>Group F: 8.14 ng/ml (0.57–69.19)<br>Statistically significant difference	PCT rose markedly during several treatment-related inflammatory states. T-cell antibody therapy, alemtuzumab, IL-2 therapy, granulocyte transfusions, and acute GvHD all produced non-infectious PCT elevations, with median peaks ranging from ~1 to 10 ng/mL and a substantial proportion of episodes reaching ⩾2.0 ng/mL. In t-cell antibody therapy, alemtuzumab, and granulocyte transfusions, PCT peaks overlapped with those seen in Gram-negative sepsis, limiting its ability to discriminate treatment-related inflammation from true infection.
Koya et al. (2012)	103 with fever after HSCT:<br>67 Non-infectious febrile<br>7 Intracellular infections<br>30 Bacterial or fungal infections	Prospective	Cut-off value 0.5 ng/mL<br>AUC_{ROC} = 0.82<br>Specificity of 100% & sensitivity of 41% for PCT < 0.25 ng/mL for first 5 days of fever<br>Statistically significant difference	PCT remained low in non-infectious post-transplant complications, including acute GvHD, CMV infection, engraftment syndrome, thrombotic microangiopathy, relapse, enterocolitis, and immune-mediated pulmonary events. On day 1 of fever, PCT was significantly lower in non-infectious episodes than in systemic bacterial or fungal infections and outperformed CRP diagnostically (AUC 0.82 vs. 0.76) using a 0.5 ng/mL cutoff. Persistently low PCT (⩽0.25 ng/mL over the first 5 days) excluded systemic bacterial or fungal infection with 100% specificity, and combining PCT >0.5 ng/mL with CRP >4 mg/dL further improved specificity and positive predictive value for identifying true infection.
Ortega et al. (2004)	92 with fever after HSCT:<br>10 Fever of other cause<br>51 Fever of unknown cause<br>31 Infectious Aetiology (26 Microbiological and 5 clinical confirmation)	Prospective	Microbiological confirmation 0.5 ± 0.7 ng/ml<br>Clinical confirmation 0.2 ± 0.2 ng/ml<br>Fever of another cause 1.7 ± 4.2 ng/ml<br>Fever of unknown cause 0.3 ± 0.2 ng/ml<br>No Statistically significant difference	Serum PCT levels on the first day of fever did not differ significantly between infectious and non-infectious etiologies. The non-infectious group—driven largely by GvHD, engraftment syndrome, capillary leak syndrome, and veno-occlusive disease—showed a wide PCT range (mean 1.7 ± 4.2 ng/mL), overlapping substantially with infectious episodes.

Blijlevens et al. (2000)	12 with fever after BMT:<br>5 non-infectious<br>7 infectious	Prospective	Patients with additional IE 0.37 ± 0.05 μg/L<br>Control group (mucositis alone) 0.11 ± 0.03 μg/L<br>Statistically significant difference	PCT levels remained low throughout the post-transplant period and never exceeded 4 ng/mL. Mucositis—present in all patients—served as the baseline inflammatory condition, and PCT showed no significant difference between mucositis alone and mucositis with an additional IE during days 1–12. Only on day 15 did PCT rise modestly in IE cases (0.37 ± 0.05 ng/mL vs. 0.11 ± 0.03 ng/mL), largely driven by GvHD.
Schüttrumpf et al. (2003)	95 with fever:<br>9 drug-related fever<br>8 tumor-associated fever<br>78 infectious	Prospective	Cutoff value 0.2 μg/L<br>AUC_{ROC} = 0.876 (0.792-0.935)<br>Sensitivity 80.8%<br>Specificity 82.4%<br>Infectious 0.7 μg/L (0.0-19.9 μg/L)<br>Drug-related fever 0.0 μg/L (0.0-0.5 μg/L)<br>Tumor-associated fever 0.2 μg/L (0.0-0.4 μg/L)<br>Statistically significant difference	PCT levels were significantly lower in non-infectious etiologies (drug-related and tumor-associated fever) compared with infectious causes or fever of unknown origin. Nearly all non-infectious cases had PCT <0.5 ng/mL. PCT demonstrated strong discriminatory ability (AUC 0.876), clearly outperforming CRP (AUC 0.654). An optimal cutoff of 0.2 ng/mL provided 80.8% sensitivity and 82.4% specificity for identifying infection
Giamarellou et al. (2004)	158 with febrile neutropenia:<br>55 FUO<br>52 Bacteraemia<br>20 Clinically localized infection<br>14 Localized bacterial infection<br>12 Severe sepsis<br>5 systemic mycosis	Prospective	FUO 1.11 ± 0.27 ng/mL<br>Bacteraemia 2.98 ± 1.03 ng/mL<br>Clinically localized infection 0.87 ± 0.23 ng/mL<br>Localized bacterial infection 0.98 ± 0.24 ng/mL<br>Severe sepsis 14.54 ± 5.05 ng/mL<br>Systemic mycosis 1.17 ± 0.44 ng/mL<br>Statistically significant difference	PCT criteria (<0.5 ng/mL indicating fever of unknown origin, FUO) did not align with clinical classifications. Despite being clinically categorized as FUO, patients had a substantially higher mean PCT level on day 1 of fever (1.11 ± 0.27 ng/mL)
Hangai et al. (2014)	91 with fever:<br>39 Tumor fever (group1)<br>26 Infection before chemotherapy (group2)	retrospective	Tumor fever 0.19 ± 0.21 ng/mL<br>Infection before chemotherapy (0.38 ± 0.39 ng/mL<br>bloodstream infection after chemotherapy 8.9 ± 28.8 ng/mL<br>(group 1 and 2):	PCT levels differed markedly between tumor fever and infectious etiologies: tumor fever showed low values (0.19 ± 0.21 ng/mL), infections before chemotherapy showed modest increases (0.38 ± 0.39 ng/mL), and severe post-chemotherapy infections displayed markedly elevated levels (8.9 ± 28.8 ng/mL). The PCT/CRP ratio provided superior diagnostic

	26 bloodstream infection after chemotherapy (group3)		Cutoff value 0.27 ng/mL<br>AUC 0.697<br>Specificity 82.1%<br>Sensitivity 50%<br>PPV 65%<br>NPV 71.1%<br>(group1 and 3):<br>Cutoff value 0.34 ng/mL<br>AUC 0.702<br>Specificity 87.2<br>Sensitivity 46.2<br>PPV 70.6%<br>NPV 70.8%<br>Statistically significant difference	discrimination (AUC 0.745), outperforming PCT and CRP alone. A ratio cutoff of 0.071 differentiated tumor fever from pre-chemotherapy infection with 53.8% sensitivity and 89.7% specificity, while a cutoff of 0.014 distinguished tumor fever from severe post-chemotherapy infection with 96.2% sensitivity and 53.8% specificity, yielding a high NPV (95.5%).
Jimeno et al. (2004)	104 undergoing chemotherapy with fever and neutropenia:<br>63 FUO<br>26 CDI<br>15 MDI	Prospective	FUO 0.21 ng/mL (0.09–1.43)<br>CDI 0.27 ng/mL (0.10–10.6)<br>MDI 1.24 ng/mL (0.09–10.9)<br>Cutoff value 0.5 ng/mL<br>Sensitivity 66.7%<br>Specificity 86.5%<br>PPV 45.5%<br>NPV 93.9%<br>Statistically significant difference	Serum PCT levels were significantly higher in microbiologically documented infections (MDI) (median 1.24 ng/mL) compared with clinically documented infections (0.27 ng/mL) and fever of unknown origin (0.21 ng/mL). Using a 0.5 ng/mL cutoff, only 13.4% of FUO/CDI episodes exceeded this threshold versus 66.7% of MDI cases. A ≥50% rise in PCT from baseline occurred far more often in MDI (50%) than in CDI (18.2%) or FUO (12%). Low PCT values in FUO and CDI reinforce its specificity for true bacterial infection.
Ciaccio et al. (2004)	54 oncohematologic patients:<br>30 bacterial infections (group A)<br>9 viral infections (group B)<br>15 Controls (group C)	Prospective	≥0.5 ng/mL:<br>Group A (27 out of 30)<br>Group B (8 out of 9)<br>Group C (12 out of 15)<br>Cutoff values 0.5 – 2 ng/mL<br>No statistically significant difference	Elevated PCT (≥0.5 ng/mL) occurred in 80% of non-infected controls—nearly identical to rates in bacterial (90%) and viral (89%) infections. PCT distributions overlapped substantially across all groups, with no significant differences at cutoffs between 0.5 and 2 ng/mL.
Matzaraki et al. (2007)	58 patients:<br>15 controls (group A)<br>21 solid tumors without metastasis (group B)	Prospective	Group A 0.284 ng/mL ± 0.039<br>Group B 0.327 ng/mL ± 0.054<br>Group C 0.690 ng/mL ± 0.225<br>Group D 1.030 ng/mL ± 0.344	Serum PCT concentrations demonstrated a stage-dependent increase, with the highest values observed in subjects with disseminated metastatic disease (1.03 ng/mL), significantly

	11 with liver metastasis only (group C)<br>11 generalized metastasis (group D)		Statistically significant difference	exceeding levels measured in healthy controls and in patients with non-metastatic solid tumors.

Table S9. Studies evaluating PCT in surgical patients

Author (Year)	Participants	Study Design	PCT Results	Key Findings
Liu et al. (2017)	251 with TAAD:<br>224 survivors<br>27 non-survivors	Prospective	Presurgery:<br>All patients: 0.11ng/mL (0.05–0.33)<br>Survivor: 0.11 ng/mL (0.52–0.32)<br>Non-survivor: 0.29 ng/mL (0.08–0.45)<br>No statistically significant difference<br>24 h postsurgery:<br>All patients: 2.73 ng/mL (0.96–9.55)<br>Survivor: 2.46 ng/mL (0.94–8.45)<br>Non-survivor: 12.38 ng/mL (2.47–37.38)<br>Statistically significant difference<br>48 h postsurgery:<br>Cutoff value 5.86 ng/mL<br>Sensitivity 70.6%<br>Specificity 74.3%<br>All patients: 1.88 ng/mL (0.67–8.05)<br>Survivor: 1.68 ng/mL (0.60–6.27)<br>Non-survivor: 19.11 ng/mL (2.63–75.31)<br>Statistically significant difference<br>7 days postsurgery:<br>All patients: 0.72 ng/mL (0.27–3.59)<br>Survivor 0.57 ng/mL (0.21–1.94)<br>Non-survivor 8.33 ng/mL (3.75–59.88)<br>Statistically significant difference	PCT rose sharply due to non-infectious surgical trauma, peaking at 24 hours postoperatively (median 2.73 ng/mL) and correlating with cardiopulmonary bypass duration. Higher early PCT levels were associated with mortality, multiple organ dysfunction, and prolonged ICU and hospital stays. A 48-hour cutoff of 5.86 ng/mL predicted death with 70.6% sensitivity and 74.3% specificity, and values >6 ng/mL identified major non-infectious complications. Reduced PCT clearance by day 7 strongly predicted poor outcomes, indicating superior prognostic value compared with absolute concentrations.
Brocca et al. (2017)	122 CS patients:<br>35 CSA-AKI	Prospective	CSA-AKI 3.3 ng/mL (0.5–7.4)<br>No AKI 0.7 ng/mL (0.3–1.7)<br>Statistically significant difference	PCT measured at 48 hours was significantly higher in those who developed CSA-AKI (median 3.3 ng/mL vs. 0.7 ng/mL). PCT

				outperformed IL-6 in predicting adverse renal outcomes, including AKI, need for renal replacement therapy, and CKD progression (AUC 0.692 vs. 0.541). PCT levels increased with the number of blood transfusions, while surgery type and extracorporeal circulation duration showed no effect on PCT concentrations.
Aouifi et al. (1999)	36 after CS:<br>12 CABG with CPB (Group 1)<br>12 CABG without CPB (Group 2)<br>12 valvular surgeries with CPB (Group 3)	Prospective	Baseline:<br>Group 1: 0.16 ng/mL (0.08)<br>Group 2: 0.13 ng/mL (0.10)<br>Group 3: 0.18 ng/mL (0.07)<br>Day 1:<br>Group 1: 1.3 ng/mL (1.8)<br>Group 2: 1.1 ng/mL (1.2)<br>Group 3: 1.4 ng/mL (1.2)<br>With SIRS: 1.79 ng/mL (1.64)<br>Without SIRS: 0.34 ng/mL (0.32)<br>Statistically significant difference	Surgery-induced SIRS produced moderate PCT elevations peaking on day 1 (≈1.1–1.4 ng/mL) and returning to baseline by day 5. Patients meeting SIRS criteria showed higher PCT levels (1.79 vs. 0.34 ng/mL), but values remained <5 ng/mL in uncomplicated postoperative courses. A PCT >5 ng/mL reliably signaled postoperative complications, including both infections and severe non-infectious events such as shock. PCT's rapid kinetics and greater specificity compared with CRP support its utility for early detection of postoperative complications.
Meisner et al. (1998)	117 surgical patients:<br>37 minor aseptic surgery (Group 1)<br>11 minor abdominal surgery (Group 2)<br>22 abdominal surgeries of intestines (Group 3)<br>16 major abdominal or thoracic surgery (group 4)<br>44 Cardiac or thoracic surgery (Group 5)	Prospective	Group 1: 0.38 µg/L<br>Group 2: 0.49 µg/L<br>Group 3: 1.5 µg/L<br>Group 4: 0.54 µg/L<br>Group 5: 0.61 µg/L<br>Statistically significant difference	PCT increased after surgery in a procedure-dependent manner: minor aseptic operations produced small rises rarely exceeding 1 µg/L, cardiac/thoracic surgery generated higher but generally <2 µg/L, and major abdominal or intestinal surgery induced the largest elevations (median up to 1.5 µg/L). Patients with postoperative complications had markedly higher PCT levels (median 6.48 µg/L). The study proposes surgery-specific PCT thresholds (>1 µg/L for minor procedures and >2 µg/L for cardiac surgery) and emphasizes interpretation based on procedure type and serial measurements.

Dörge et al. (2003)	80 after cardiac surgery on CPB:<br>27 infectious<br>47 with complications (20 non-infectious and 27 infectious)<br>33 without complications	Prospective	Preoperative PCT <0.2 ng/mL<br>AUC 0.720 (0.603-0.837)<br>Post-operative PCT:<br>With complications 30.3 ± 6.7 ng/ml<br>Without complications 5.5 ± 1.4 ng/ml<br>Infectious 38.4 ± 11.3 ng/ml<br>Non-infectious 10.8 ± 1.6 ng/ml<br>Statistically significant difference	Postoperative PCT rose markedly despite normal preoperative levels. Non-survivors and patients with severe complications had significantly higher PCT than survivors and uncomplicated cases (e.g., 34.3 vs. 15.9 ng/mL). PCT elevations did not differ between infectious and non-infectious complications, indicating that CPB-induced SIRS—rather than bacterial infection—was the primary driver of postoperative PCT increases.
Bianchi et al. (2006)	31 after elective cephalic pancreatoduodenectomy:<br>6 without complications<br>24 with complications (21 non-deceased and 3 deceased)<br>1 Excluded	Prospective	Without complications: 84% > 2ng/mL on the first post operative day, 84% and 67% >0.5ng/mL 2nd and 3rd days, finalizing with normal values on the 4th and 5th post operative day in 100% of the patients.<br>With complications: 86% >2ng/mL on the first 2 days, 76% and 57% with > 0.5ng/mL on 3rd and 4th day and 57% with normal values on the 5th day post-operative	PCT levels were consistently higher in complicated than in uncomplicated postoperative courses. Uncomplicated cases normalized PCT to <0.5 ng/mL by days 4–5, whereas complicated cases often maintained PCT >2 ng/mL, with fatal outcomes showing persistently high values (≥10 ng/mL). Persistent postoperative elevation suggested sustained inflammatory activation and possible endotoxin translocation.

Table S10. Studies evaluating PCT in traumatology

Author (Year)	Participants	Study Design	PCT Results	Key Findings
He et al. (2017)	251 with ICH:<br>161 unfavorable outcomes<br>51 deceased	Prospective	Total: 0.053 ng/mL (0.035–0.078)<br>Mortality:<br>0.072 µg/L (0.045–0.086)<br>Cutoff value 0.555 ng/mL<br>Sensitivity 66.7%<br>Specificity 58.5%<br>AUC 0.652 (0.569–0.735)<br>Unfavorable outcome:<br>0.065 ng/mL (0.044–0.087)<br>Sensitivity 74.5%<br>Specificity 56.7%<br>AUC 0.701 (0.635–0.767)	Even mild non-infectious PCT elevation at admission (median 0.053 µg/L) independently predicted poor 3-month outcomes and mortality. Patients in the highest PCT quartile (>0.078 µg/L) had over sevenfold higher odds of unfavorable functional outcome (OR 7.34) and death (OR 7.48). PCT outperformed hs-CRP and leukocyte count as a prognostic marker and correlated with hematoma and perihematomal edema volumes, linking systemic inflammatory activation to ICH severity and prognosis.

			Statistically significant difference	
Halvorson et al. (2017)	73 with intracranial hemorrhage and fever:<br>36 infectious<br>37 non-infectious	Prospective	Non-infectious 0.09 ng/mL (0.05-0.45 ng/mL)<br>Infectious 0.15 ng/mL (0.06-0.5 ng/mL)<br>No statistically significant difference	Serum PCT did not differentiate infectious from non-infectious causes. Median PCT levels were similar between infectious (0.15 ng/mL) and non-infectious fever (0.09 ng/mL), with no significant differences across hemorrhage subtypes. Multivariate analysis confirmed the lack of discriminatory value. Non-infectious fever etiologies included central hyperthermia from neurologic injury, drug reactions, venous thromboembolism, and transfusion-related responses.

Table S11. Studies evaluating PCT in transplanted patients

Author (Year)	Participants	Study Design	PCT Results	Key Findings
Hammer et al. (1998)	78 heart and lung transplanted patients:<br>59 no rejection and no infection<br>19 rejections	Retrospective	No rejection and no infection 0.3 ± 0.3 ng/mL<br>Rejection, no infection 0.2 ± 0.2 ng/mL<br>Rejection and infection 0.6 ± 0.6 ng/mL<br>Systemic infection 10.5 ± 18.2 ng/mL<br>Cut-off point <0.8 ng/mL<br>Specificity 82%<br>Sensitivity 100%<br>Statistically significant difference	PCT remained low during non-infectious inflammatory states—including acute rejection, surgical trauma, immunosuppression, viral infections, and localized infections—showing mean values around 0.2–0.3 ng/mL. In contrast, systemic infections produced markedly elevated PCT levels (mean 10.5 ng/mL). A cutoff of 0.8 ng/mL distinguished systemic infection from acute rejection with excellent diagnostic accuracy (100% sensitivity; 82% specificity), highlighting PCT's high specificity for systemic bacterial infection in transplant populations.
Boeken et al. (2000)	74 patients:<br>60 CABG:<br>30 without postoperative complications (group A)<br>15 postoperative sepsis (group B)<br>15 SIRS (group C)	Prospective	Intraoperative:<br>Group A < 0.2 ng/ml<br>2nd day postoperative:<br>Group A 0.33 ± 0.15 ng/ml<br>Group B 19.6 ± 6.2 ng/ml<br>Group C 0.7 ± 0.4ng/ml<br>Endomyocardial biopsy:	PCT remained low (<0.2 ng/mL) during uncomplicated postoperative courses, SIRS, rejection episodes, and viral infections, indicating that these conditions do not trigger significant PCT elevation. In contrast, PCT rose sharply in bacterial sepsis after CABG (mean 19.6 ng/mL, peaking on day 2) and was significantly elevated in bacterial or

	14 heart Transplant (65 biopsy):<br>8 with rejection Group I<br>7 with viral infection Group II<br>4 bacterial or fungal infection Group III<br>Control Group IV		Group I 0.18 ± 0.06 ng/ml<br>Group II 0.30 ± 0.09 ng/ml<br>Group III 1.63 ± 1.16 ng/ml<br>Group IV 0.21 ± 0.09 ng/ml<br>Statistically significant difference	fungal infections among HTX recipients (mean 1.63 ng/mL), while remaining low in non-infectious or viral states (0.2–0.3 ng/mL). These findings support PCT as a specific early marker for differentiating bacterial/fungal infection from rejection, viral infection, or sterile inflammation in cardiac surgery and heart-transplant populations.
Kuse et al. (2000)	40 after liver transplant:<br>11 infectious complications<br>11 rejections	Prospective	Post-surgery 5.2 ± 1.23 ng/mL (1.2-15.5)<br>Infectious ≥ 0.8 ng/mL<br>Statistically significant difference	PCT reliably differentiated infection from acute rejection. Although PCT rose postoperatively due to surgical trauma (mean peak 5.2 ng/mL), levels did not increase during 11 episodes of acute rejection and often declined from postoperative baselines. In contrast, all 11 infection episodes showed marked PCT elevation (≥0.8 ng/mL), with the highest values seen in systemic intra-abdominal infections (7.9–41 ng/mL). PCT correlated with infection severity and decreased in response to effective treatment, supporting its utility in distinguishing infection from rejection.
Shim et al. (2021)	91 with fever within 1-month post-pancreas-transplant:<br>46 infectious<br>45 non-infectious	Retrospective	Non-infectious 0.26 ± 0.26 ng/ml<br>Infectious 0.94 ± 1.07 ng/ml<br>Statistically significant difference	Peak serum PCT levels were significantly higher in infectious than non-infectious etiologies (0.94 ± 1.07 vs. 0.26 ± 0.26 ng/mL). Multivariate analysis showed PCT outperformed CRP for distinguishing infectious from non-infectious fever. Non-infectious causes—including hematoma, transfusion reactions, rejection, contrast reactions, and unexplained fevers—generally showed PCT concentrations below the optimal diagnostic cutoff of 0.405 ng/mL.
Kuse et al. (2000)	40 after liver transplantation:<br>16 uneventful<br>11 infectious<br>11 rejections	Prospective	Infectious ≥ 0.8 ng/mL (7,9 – 41)<br>Uneventful 5.2 ng/mL (1.2 -15.5)<br>AUC 0.93<br>Cutoff value 5.9 ng/mL<br>Sensitivity 100%<br>Specificity 75%<br>Statistically significant difference	PCT rose transiently after surgical trauma (mean peak 5.2 ng/mL) and normalized within one week in uncomplicated cases. Acute rejection did not elevate PCT, whereas infection episodes produced marked increases; a cutoff of 5.9 ng/mL distinguished infection from rejection with excellent accuracy (100% sensitivity; 75% specificity; AUC 0.93). PCT

				correlated with disease-severity scores, and persistently high levels (>10 ng/mL) signaled poor outcomes and inadequate therapeutic response.
Fazakas et al. (2003)	61 underwent liver transplant:<br>32 without complications (group A)<br>29 with complications (Group B):<br>13 with sepsis<br>14 without sepsis	Prospective	HV 1.27 ± 0.43 ng/mL<br>Systemic sample 0.16 ± 0.26 ng/mL<br>PV 0.23 ± 0.15 ng/mL<br>Group A 4.8 ± 3.6 ng/mL<br>Group B 30.6 ± 19.6 ng/mL<br>Statistically significant difference<br>Group B Day 2:<br>With sepsis 30.89 ± 18.32 ng/mL<br>Without sepsis 30.5 ± 15.4 ng/mL<br>No statistically significant difference	PCT increased significantly after graft reperfusion (from 0.27 to 1.04 ng/mL), with hepatic-vein concentrations markedly higher than systemic and portal levels, indicating intrahepatic PCT production during non-infectious injury. By postoperative day 2, patients with major complications exhibited substantially elevated systemic PCT (30.6 ng/mL) compared with those without major complications (4.8 ng/mL), independent of sepsis. PCT correlated with APACHE II scores (r = 0.58), while elevated donor PCT levels showed no association with recipient infection or outcomes.
Wagner et al. (2001)	79 heart donors:<br>8 deceased within 30 days<br>71 non-deceased	Retrospective	Deceased 1.7 ng/mL (0.05-5.32)<br>Non-deceased 0.25 ng/mL (0.05-7.28)<br>Cutoff value >2ng/mL<br>Sensitivity 50%<br>Specificity 95.8%<br>AUC 0.71<br>Statistically significant difference	PCT levels were significantly higher in donors whose recipients died from early graft failure (median 1.7 ng/mL) compared with donors whose recipients survived (median 0.25 ng/mL). A cutoff >2 ng/mL predicted early graft-failure–related mortality with high specificity (95.8%) but moderate sensitivity (50%). Elevated donor PCT independently predicted recipient mortality (adjusted OR 43.8) and outperformed CRP (AUC 0.71 vs. 0.64).

Supplementary File S1 – Full electronic search strategies.

Databases searched: PubMed/MEDLINE; Embase; Web of Science Core Collection; Scopus.
Coverage: database inception through 31 July 2025.
Limits applied across databases: Humans; English language.
Final search date: 31 July 2025.

PubMed/MEDLINE (Humans; English; inception–31 July 2025)
("Procalcitonin"[MeSH Terms] OR procalcitonin[Title/Abstract] OR PCT[Title/Abstract]) AND (noninfect*[Title/Abstract] OR "non-infect*"[Title/Abstract] OR "sterile inflammation"[Title/Abstract] OR inflammation[Title/Abstract] OR trauma*[Title/Abstract] OR shock[Title/Abstract] OR "cardiogenic shock"[Title/Abstract] OR surg*[Title/Abstract] OR postoperative[Title/Abstract] OR "post-operative"[Title/Abstract] OR pancreatitis[Title/Abstract] OR burn*[Title/Abstract] OR malignan*[Title/Abstract] OR cancer[Title/Abstract] OR neoplasm*[Title/Abstract] OR autoimmune[Title/Abstract] OR rheumat*[Title/Abstract] OR "organ failure"[Title/Abstract] OR "renal failure"[Title/Abstract] OR "kidney failure"[Title/Abstract] OR dialysis[Title/Abstract] OR hemodialysis[Title/Abstract] OR transplant*[Title/Abstract] OR rejection[Title/Abstract])

Embase (Humans; English; inception–31 July 2025)
('procalcitonin'/exp OR procalcitonin:ti,ab OR pct:ti,ab) AND (noninfect*:ti,ab OR 'non infect*':ti,ab OR 'sterile inflammation':ti,ab OR inflammation:ti,ab OR trauma*:ti,ab OR shock:ti,ab OR 'cardiogenic shock':ti,ab OR surg*:ti,ab OR postoperative:ti,ab OR 'post operative':ti,ab OR pancreatitis:ti,ab OR burn*:ti,ab OR malignan*:ti,ab OR cancer:ti,ab OR neoplasm*:ti,ab OR autoimmune:ti,ab OR rheumat*:ti,ab OR 'organ failure':ti,ab OR 'renal failure':ti,ab OR 'kidney failure':ti,ab OR dialysis:ti,ab OR hemodialysis:ti,ab OR transplant*:ti,ab OR rejection:ti,ab) AND [english]/lim AND [humans]/lim

Web of Science Core Collection (English; inception–31 July 2025)
TS=(procalcitonin OR PCT) AND TS=(noninfect* OR "non-infect*" OR "sterile inflammation" OR inflammation OR trauma* OR shock OR "cardiogenic shock" OR surg* OR postoperative OR "post-operative" OR pancreatitis OR burn* OR malignan* OR cancer OR neoplasm* OR autoimmune OR rheumat* OR "organ failure" OR "renal failure" OR "kidney failure" OR dialysis OR hemodialysis OR transplant* OR rejection)

Scopus (English; inception–31 July 2025)
TITLE-ABS-KEY(procalcitonin OR pct) AND TITLE-ABS-KEY(noninfect* OR "non-infect*" OR "sterile inflammation" OR inflammation OR trauma* OR shock OR "cardiogenic shock" OR surg* OR postoperative OR "post-operative" OR pancreatitis OR burn* OR malignan* OR cancer OR neoplasm* OR autoimmune OR rheumat* OR "organ failure" OR "renal failure" OR "kidney failure" OR dialysis OR hemodialysis OR transplant* OR rejection) AND LIMIT-TO(LANGUAGE, "English")
